# Regulation of Mutagenic DNA Polymerase V Activation in Space and Time

**DOI:** 10.1371/journal.pgen.1005482

**Published:** 2015-08-28

**Authors:** Andrew Robinson, John P. McDonald, Victor E. A. Caldas, Meghna Patel, Elizabeth A. Wood, Christiaan M. Punter, Harshad Ghodke, Michael M. Cox, Roger Woodgate, Myron F. Goodman, Antoine M. van Oijen

**Affiliations:** 1 Zernike Institute for Advanced Materials, Centre for Synthetic Biology, University of Groningen, Groningen, The Netherlands; 2 Laboratory of Genomic Integrity, National Institute of Child Health and Human Development, National Institutes of Health, Bethesda, Maryland, United States of America; 3 Departments of Biological Sciences and Chemistry, University of Southern California, Los Angeles, California, United States of America; 4 Department of Biochemistry, University of Wisconsin-Madison, Madison, Wisconsin, United States of America; Northeastern University, UNITED STATES

## Abstract

Spatial regulation is often encountered as a component of multi-tiered regulatory systems in eukaryotes, where processes are readily segregated by organelle boundaries. Well-characterized examples of spatial regulation are less common in bacteria. Low-fidelity DNA polymerase V (UmuD′_2_C) is produced in *Escherichia coli* as part of the bacterial SOS response to DNA damage. Due to the mutagenic potential of this enzyme, pol V activity is controlled by means of an elaborate regulatory system at transcriptional and posttranslational levels. Using single-molecule fluorescence microscopy to visualize UmuC inside living cells in space and time, we now show that pol V is also subject to a novel form of spatial regulation. After an initial delay (~ 45 min) post UV irradiation, UmuC is synthesized, but is not immediately activated. Instead, it is sequestered at the inner cell membrane. The release of UmuC into the cytosol requires the RecA* nucleoprotein filament-mediated cleavage of UmuD→UmuD′. Classic SOS damage response mutants either block [*umuD*(K97A)] or constitutively stimulate [*recA*(E38K)] UmuC release from the membrane. Foci of mutagenically active pol V Mut (UmuD′_2_C-RecA-ATP) formed in the cytosol after UV irradiation do not co-localize with pol III replisomes, suggesting a capacity to promote translesion DNA synthesis at lesions skipped over by DNA polymerase III. In effect, at least three molecular mechanisms limit the amount of time that pol V has to access DNA: (1) transcriptional and posttranslational regulation that initially keep the intracellular levels of pol V to a minimum; (2) spatial regulation via transient sequestration of UmuC at the membrane, which further delays pol V activation; and (3) the hydrolytic activity of a recently discovered pol V Mut ATPase function that limits active polymerase time on the chromosomal template.

## Author Summary

*Escherichia coli*, and many other bacteria, respond to high levels of DNA damage with an inducible system called the SOS response. In this response, bacteria first try to restart replication using non-mutagenic DNA repair strategies. If that fails, replication can be restored using DNA polymerases that simply replicate over DNA lesions, a desperation strategy that results in mutations. DNA polymerase V (pol V) is responsible for most mutagenesis that accompanies the SOS response. Because of the risk inherent to elevated mutation levels, pol V activation is tightly constrained. This report introduces a new layer of regulation on pol V activation, with a novel spatial component. After synthesis, the UmuC subunit of pol V is sequestered transiently at the membrane. Release into the cytosol and final activation depends on the activity of RecA protein and the autocatalytic cleavage of UmuD to generate the UmuD' subunit of pol V. The resulting delay in activation represents an additional molecular mechanism that limits the amount of time that this sometimes necessary but potentially detrimental enzyme spends on the DNA.

## Introduction

Replication of the *Escherichia coli* chromosome is carried out by replisomes: dynamic multi-protein complexes that coordinate genome duplication by DNA polymerase (pol) III [1]. Pol III replisomes are both exceptionally fast [1] and accurate [2], but are inefficient at synthesising DNA on damaged templates [3]. High levels of DNA damage lead to replication-fork collapse, which can be lethal if not resolved. This situation is usually addressed by the approximately 40 genes of the bacterial SOS response, induced in two stages that reflect two different strategies for restoring replication. The earliest stage features induction of proteins involved in several pathways of error-free DNA repair. If this initial repair does not suffice to restart DNA replication, a later mutagenic process ensues with the induction of the *umuDC* operon [4]. This operon encodes the translesion synthesis polymerase, pol V [5]. Pol V is responsible for UV- and most chemical-induced chromosomal mutagenesis [6]. SOS mutagenesis also results in more rapid adaptation to stress and development of resistance to antibiotics in the absence of exogenous DNA damage [7,8]. Translesion DNA synthesis by pol V allows for resumption of replication on heavily damaged chromosomes, but dramatically increases mutation rates. Pol V is activated only after the cell’s capacity for non-mutagenic DNA repair has been exceeded. An elaborate regulatory regime should not be surprising, but the cellular constraints on pol V activity remain imperfectly understood.

**Fig 1:**
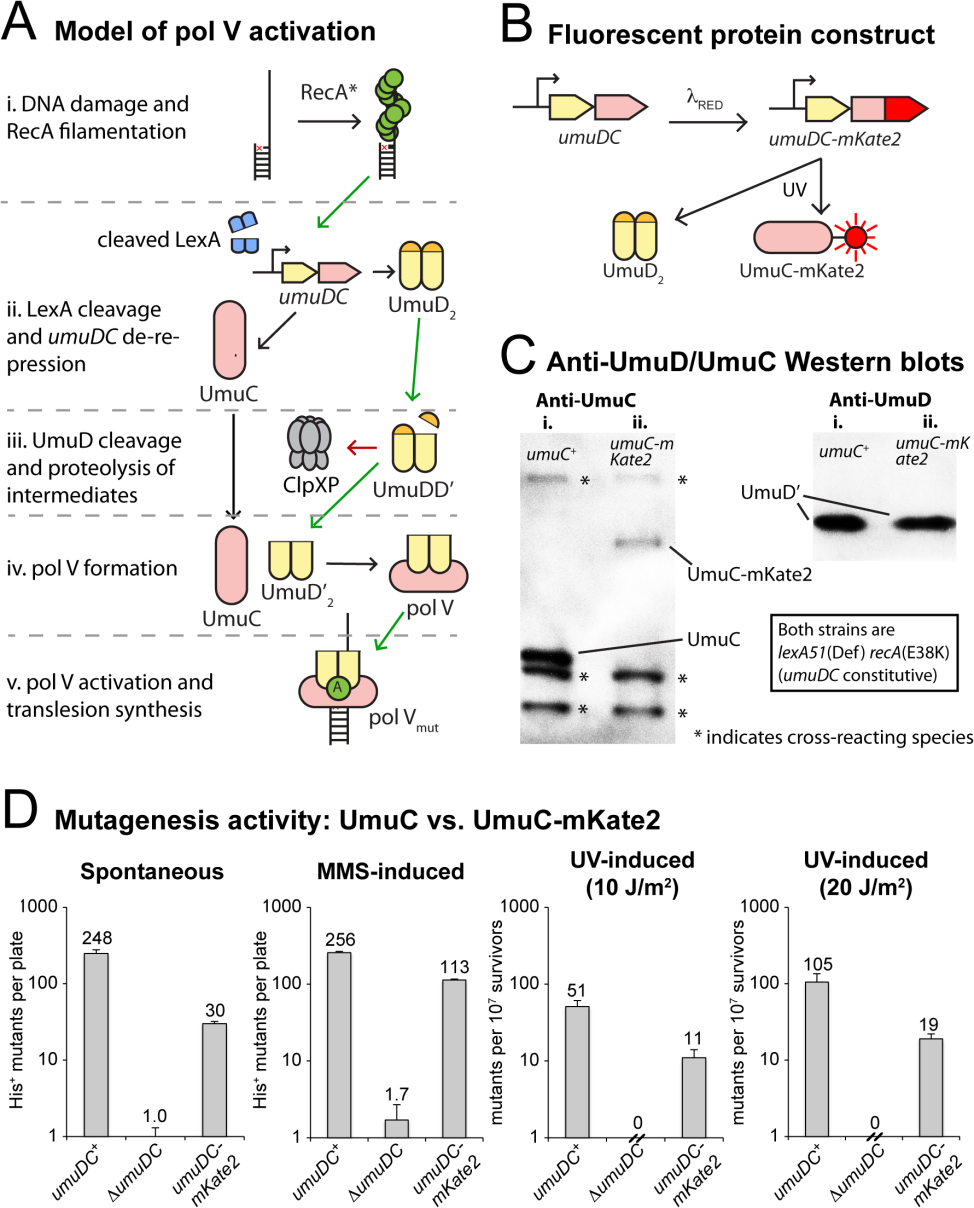
Construction of a fluorescent UmuC reporter strain to visualize pol V regulation. (A) Sketch depicting the regulation of pol V activity in *E*. *coli*. Transcriptional repression, targeted proteolysis, posttranslational modification and protein-protein interactions regulate levels of pol V Mut (UmuD’_2_-UmuC-RecA-ATP), which is capable of mutagenic translesion DNA synthesis. The symbol “A” represents a molecule of ATP bound to RecA in the activated pol V Mut complex. (B) Replacement of the wild-type chromosomal *umuC* locus with *umuC-mKate2* using λ_RED_ recombineering. (C) Western blots with anti-UmuC and anti-UmuD antibodies confirming the expression of full length UmuC-mKate2. Note that steady-state levels of the chimeric UmuC-mKate2 protein (lane ii, *umuC-mKate2* background, strain RW1314) are approximately 20% of the wild-type UmuC protein (lane i, wild-type *umuC* background, RW574). Bands resulting from cross-reaction of the anti-UmuC antibody (see S1A Fig) with other proteins are also observed (marked with *) and indicate that similar amounts of lysate were loaded in each lane. Both strains are *lexA*(Def) *recA*(E38K) and thus constitutively express UmuD and UmuC and convert UmuD to UmuD'. Steady-state levels of UmuD' are similar in the wild-type *umuC* ^+^ (lane i) and *umuC-mKate2* cells (lane ii). (D) *In vivo* mutagenesis assays performed on RW1314 show that UmuC-mKate2 is active for both spontaneous and damage-induced mutagenesis. Levels of mutagenesis are lower than that of the wild-type UmuC strain (RW574), but are consistent with overall lower steady state levels of the chimera.

The active form of the enzyme, pol V Mut, is produced through a series of steps dependent on nucleoprotein filaments of RecA, denoted as RecA*, that are formed on single-stranded DNA after damage (**Fig 1A**). The *umuDC* operon, which encodes the pol V precursors UmuD_2_ and UmuC, contains a particularly high-affinity binding site for the LexA repressor that limits transcription of the *umuDC* genes [9]. Intracellular levels of UmuD and UmuC are further kept to a minimum through Lon-mediated proteolytic degradation [10]. As a result UmuD and UmuC only accumulate approximately 30 min after DNA damage [4]. However, UmuD_2_ and UmuC are not active for DNA synthesis. UmuD_2_ must first undergo a RecA*-mediated autocatalytic cleavage reaction that removes the N-terminal 24 amino acid residues of each subunit to generate UmuD′_2_. However, this reaction is inefficient and leads to the formation of UmuD/D' heterodimers. UmuD' in the UmuD/D' heterodimer is then rapidly targeted for degradation by the ClpXP protease [10]. As a consequence, UmuD' homodimers only accumulate in response to a persistent damage-inducing signal. UmuD'_2_ then associates with UmuC to form pol V (UmuD′_2_C) [5]. Pol V will only accumulate if the damage, and thus RecA*, persists until after the initial 45 minutes of error-free repair [4]. Pol V has weak catalytic activity *in vitro* [5,11] and is unable to promote translesion DNA synthesis in the absence of RecA *in vivo* [12]. To facilitate translesion synthesis, pol V must physically interact with RecA* and remove a single RecA-ATP molecule from the 3'-proximal end of the filament to form the mutagenic and highly active pol V Mut (UmuD'_2_-UmuC-RecA-ATP) [13,14].

Direct observation of the various activation steps of pol V inside living cells with fluorescence microscopy would allow for the testing of various hypotheses and potentially the construction of a global model for mutasome activity. However, analysis of pol V activity *in vivo* has long proven difficult due its very low expression levels [15]. Here, we report the use of single-molecule fluorescence microscopy to directly visualize the dynamics of pol V inside living cells. Surprisingly, we observe that pol V precursors are temporarily sequestered at the cellular membrane, adding another prominent factor to those limiting pol V activity during early stages of the DNA damage response. Spatial regulation of a DNA processing enzyme is unprecedented in bacteria and suggests the presence of several layers of spatiotemporal partitioning to regulate the molecular processes underlying genomic maintenance.

## Results

### Single-molecule observation of pol V in live *E*. *coli* cells

In order to visualize the regulation of pol V, we altered the *umuDC* operon so that the bright red fluorescent protein mKate2 is fused to the C-terminus of UmuC (**Fig 1B)**. Western blotting of the chromosomally expressed UmuC-mKate2 protein revealed expression of the chimeric protein, albeit at steady-state levels somewhat lower than untagged UmuC (**Fig 1C, S1A Fig**). Nevertheless, the chimeric *umuC*-mKate2 allele promoted both spontaneous and damage-induced reversion of the *hisG4* allele, confirming it is functionally active (**Fig 1D, S1B Fig**). The level of mutagenesis promoted by UmuC-mKate2 was lower than that of wild-type UmuC, which is consistent with the overall lower steady-state levels of the chimera in the cell.

We imaged *E*. *coli* K12 MG1655 UmuC-mKate2 cells on a home-built wide-field single-molecule fluorescence microscope. To monitor changes in the cellular levels and location of UmuC as a function of time after UV-induced damage, *E*. *coli* was immobilized inside flow cells and a series of time-lapse images were recorded. We developed a novel flow-cell design that allowed for imaging of individual cells with single-molecule sensitivity, while also providing space for cells to grow into filaments (a hallmark of the SOS response) and allowing for *in situ* UV irradiation. Cells were irradiated with UV light from a mercury lamp (fluence = 1–100 J/m^2^, λ = 254 nm) and UmuC-mKate2 fluorescence was recorded over a course of 3h, imaging once every 5 min.

**Fig 2:**
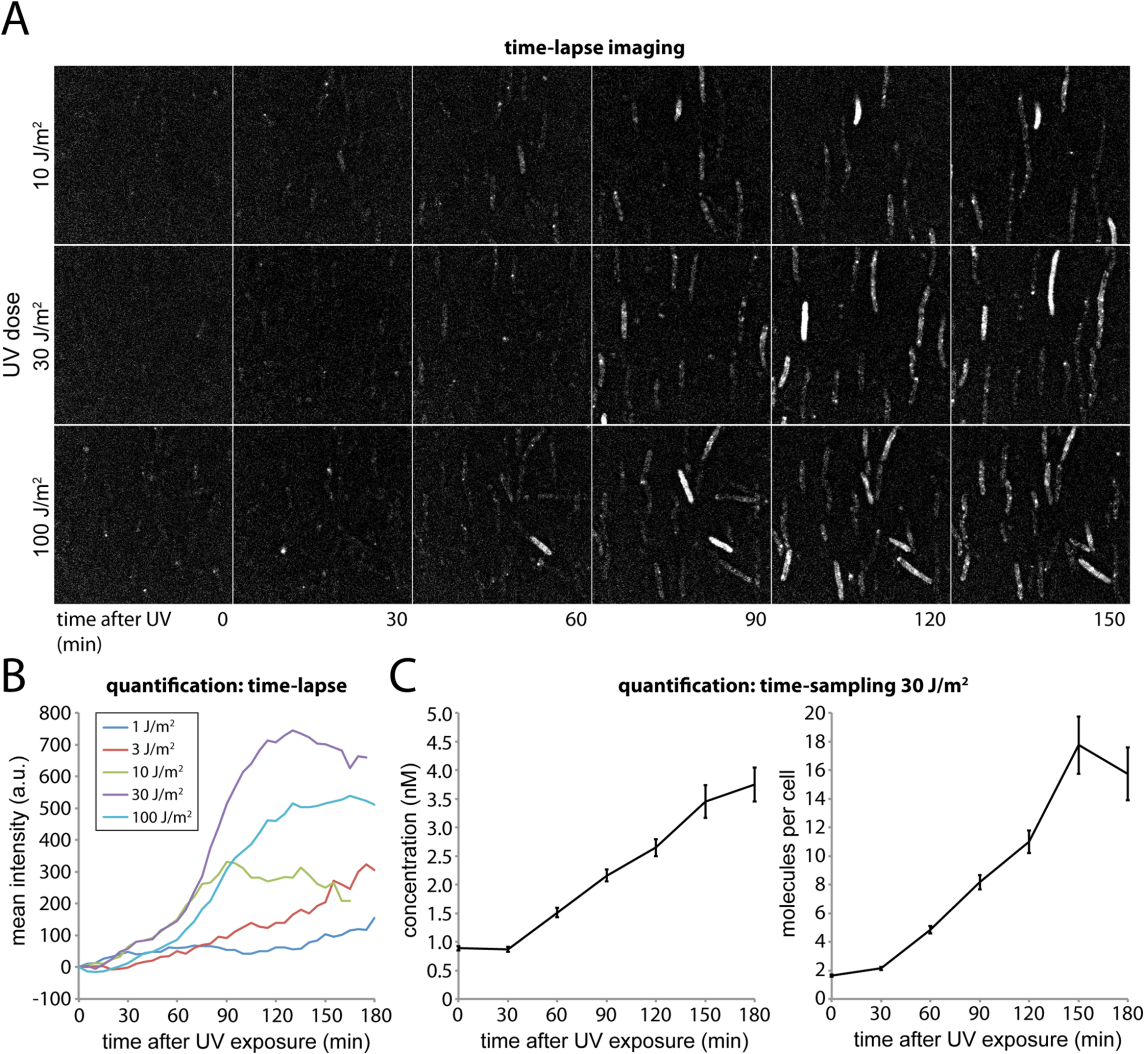
Monitoring DNA damage induced expression of UmuC-mKate2 using time-lapse and time-sampling analysis. (A) Time-lapse imaging of UmuC-mKate2 expressing cells (EAW282). Cells were grown at 37°C in flow cells and irradiated *in situ* with 10, 30 or 100 J.m^-2^ of UV light (λ = 254 nm). Following irradiation fluorescence images were recorded every 5 min for 180 min. These images reveal that UmuC induction begins around 60 mins after DNA damage. (B) Quantification of the mean fluorescence signal of cell-containing regions within time-lapse image series after various does of UV-light. (C) Quantification of the mean number of molecules per cell and mean concentration of UmuC-mKate2 in time-sampling measurements after treatment with 30 J/m^2^ UV light.

We first measured changes in fluorescence intensity using time-lapse measurements, allowing us to monitor the production of UmuC-mKate2 (**Fig 2A and 2B**). At all UV doses, very little expression of UmuC-mKate2 was observed during the first 30 min following UV irradiation (**Fig 2A and 2B**), in good agreement with the previously measured value of 30 min required for expression of UmuC and UmuD [4]. Furthermore, UmuC-mKate2 levels increased at all doses after this initial delay, albeit with different kinetics. While at doses ≤ 3 J/m^2^, UmuC-mKate2 levels gradually increased throughout the duration of the experiment; at higher doses the protein levels decreased after reaching a maximum at 90–120 min. At the highest dose, 100 J/m^2^, UmuC-mKate2 was produced more slowly than at 30 J/m^2^ and ultimately reached lower levels, presumably due to inhibition of protein synthesis as the result of extensive DNA damage.

The amount of UmuC-mKate2 inside cells was quantified using time-sampling measurements (**Fig 2C**). Similar to the time-lapse experiments, cells were grown, irradiated and imaged within flow cells. Rather than periodically recording single fluorescence images to monitor the response of the same set of individual cells in time, we recorded video-rate movies of UmuC-mKate2 fluorescence using a new population of cells for every time point after UV irradiation. This procedure provided higher-quality intensity information but led to significant photobleaching. We therefore chose a new field of view with unbleached cells for each new measurement. Movies (296 × 34 ms frames) were recorded over 10 fields of view every 30 min for 3 h, capturing between 70 and 236 cells. For each field of view we also recorded a bright-field image, allowing us to define the outline of each cell. The fluorescence movies were used to determine the concentration of UmuC-mKate2 inside each cell **(S2 Fig)**.

Plotting the average cellular concentration of UmuC-mKate2 over time, we observed a sharp increase (**Fig 2C**), with dynamics consistent with those measured by time-lapse measurements (**Fig 2B**). At 30 J/m^2^, the concentration of UmuC-mKate2 increased from 0.9 ± 0.04 nM (S.E.M, *n* = 236 cells) to 3.7 ± 0.3 nM (*n* = 70), 180 min after irradiation with UV light. These concentrations correspond to an increase from an average of 1.6 ± 0.08 (*n* = 236) molecules per cell to 15.7 ± 1.8 (*n* = 70) molecules per cell at 180 min. The numbers of UmuC molecules measured by fluorescence microscopy (16 per cell) are lower than measurements obtained by Western blotting of wild-type cells with anti-UmuC antibodies (~ 60 per cell) [4, 15], but again, this observation is consistent with overall lower intracellular steady-state levels of the UmuC-mKate2 chimera compared to the wild-type UmuC protein (**Fig 1C**). Quantification of UmuC-mKate2 levels in cells periodically sampled from shaking culture showed similar expression dynamics and concentration levels as cells grown in flow cells: no UmuC-mKate2 is produced during the first 30 min, then levels rise to 3 nM in the period 40–120 min after irradiation (**S3 Fig**).

### UmuC appears at the cell membrane and is then released into the cytosol

**Fig 3:**
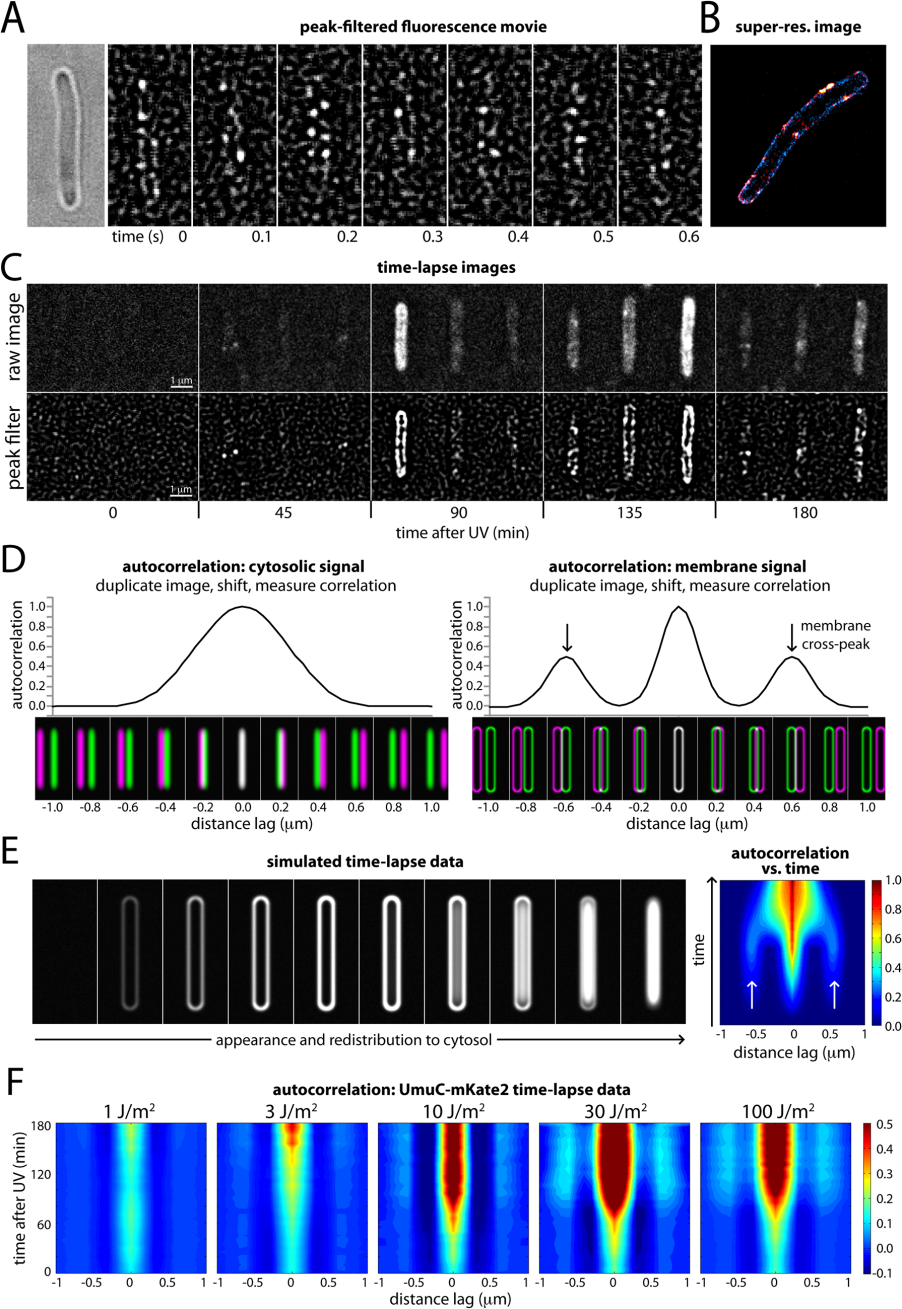
Changes in the cellular location of UmuC in response to UV irradiation. (A) Montage of frames from a time-sampling movie showing UmuC-mKate2 diffusing along the cell periphery (EAW282). A peak-enhancing filter was applied to fluorescence images [57]. (B) Two-color super-resolution reconstruction showing co-localization of UmuC-mKate2 (red) and LacY-eYFP (blue) on the cell membrane (EAW191 containing pBAD-LacY-eYFP). (C) Individual cells cropped from time-lapse series showing UmuC transitioning from a membrane-associated to a cytosolic localization (EAW282). (D) Autocorrelation analysis of simulated images of *E*. *coli* cells displaying cytosolic and membrane-associated signals. Autocorrelation measurement can be conceptualised as follows: correlations are measured between an image and its distance-shifted duplicate. Correlations are measured as a function of shift distance (or distance lag). Autocorrelation analysis of cells displaying cytosolic signals produces a broad origin peak, whereas cells with membrane-associated signal produce a distinctive cross-peak (indicated with arrows). (E) Autocorrelation analysis of a simulated time-lapse series for an *E*. *coli* cell expressing a redistributing fluorescent protein. The simulation begins with no fluorescent protein signal. The protein is then induced, producing a membrane-associated signal before redistributing to the cytosol. Here the autocorrelation analysis is presented as a 2D contour plot. Blue areas indicate low correlation, whereas red areas indicate high correlation. Arrows indicate the membrane cross-peak that is visible when the cell has membrane-associated signal. (F) Similar autocorrelation analysis of experimentally acquired time-lapse series showing dose-dependent changes in UmuC localization (EAW282). At higher doses of UV the membrane cross-peaks begin to decline at ~90 min post-irradiation, while the origin peak becomes broader, indicating redistribution of UmuC-mKate2 into the cytosol. A peak-enhancing filter was applied to fluorescence images prior to autocorrelation analysis [57].

The fast time-sampling movies showed foci of UmuC-mKate2 that move within a thin band along the cell periphery (**Fig 3A, S1 movie**). This observation suggested that many molecules of UmuC associate with the cell membrane, diffusing slowly enough to result in sharply defined foci on a single image (34 ms frame duration), but sufficiently mobile to show movement on longer timescales. To examine this apparent membrane association further, we used a plasmid encoding a fluorescently labelled inner membrane protein (LacY-eYFP) as a reference for the position of the membrane. Super-resolution images were produced for both UmuC-mKate2 and LacY-eYFP, which revealed that many of the UmuC-mKate2 foci were indeed located with LacY on the cell membrane (**Fig 3B**). Control experiments with cells expressing free mKate2 (including the same linker as the UmuC-mKate2 construct) confirmed that membrane association is a property of UmuC, as opposed to mKate2 or the linker region (**S4 Fig**).

In both the time-lapse and time-sampling datasets, visual inspection of the movies indicated a change in the localization behaviour of UmuC during the course of the DNA-damage response. For the first 90–120 min after UV exposure, UmuC-mKate2 was predominantly located at the cell periphery, while in later images UmuC-mKate2 was distributed throughout the cytosol (**Fig 3C, S5 Fig**).

We developed a novel autocorrelation-based approach to quantify the localization behaviour of UmuC across all cells in the time-lapse measurements. Autocorrelation of images can be conceptualized as follows. First, the cell image to be analyzed is duplicated and superimposed with the original (schematically depicted in **Fig 3D**) so that the intensities of each pixel in the two images are perfectly correlated. One of the images is then shifted sideways in increments (presented in the figures as the distance lag) relative to the other image and the correlation between pixel values is re-calculated. This procedure is repeated over a range of different shift sizes (or distance lags) to produce an autocorrelation function.

The highest correlation of pixels is seen when the duplicate images are perfectly superimposed (zero lag). If significant amounts of UmuC are concentrated in the membranes, additional signal peaks will be seen at two non-zero shift increments where the right membrane of one image passes over the left membrane of the other, and vice versa (schematically shown in **Fig 3D**). Cytosolic UmuC with its distribution centered along the central cellular axis should not generate such secondary peaks.

To illustrate this approach, we simulated images of *E*. *coli* cells with a width of 0.6 μm, expressing cytosolic or membrane-localized fluorescent proteins. Cells containing cytosolic signal gave rise to an autocorrelation function with a single, broad peak at zero lag (**Fig 3D**). Cells with membrane-associated fluorescence instead produced a narrow peak at zero lag (perfect superimposition), as well as two distinctive cross peaks when the duplicate images were shifted 0.6 μm, arising from correlations between signals on the two sides of the cell.

To test if this autocorrelation approach could be used to monitor changes over time in cellular distribution of UmuC-mKate2, we simulated a time-lapse series with an *E*. *coli* cell transitioning from a state with no signal, to a membrane-associated signal, to a cytosolic signal (**Fig 3E; left**). We graphed autocorrelation functions measured at each time point of the simulation as a 2D contour plot, with blue-to-red coloring indicating low-to-high levels of correlation (**Fig 3E; right**). At each time point, autocorrelation functions were normalized to the fluorescence intensity of the cell. The amplitude of the central peak (zero lag) thus reflects relative changes in fluorescent protein concentration: the amplitude is low (blue) at lower concentrations and high (red) at high cellular concentrations. When present, membrane-bound fluorescent protein gives rise to weaker secondary signals (light blue) to either side of the central peak. Analysis of experimentally acquired data produced similar results: time-lapse images of LacY-eYFP (an inner membrane protein) produced an autocorrelation plot with a strong central peak and weaker secondary peaks, while DnaX-YPet (which is cytosolic/nucleoid associated) produced only a strong central peak (**S6 Fig**). Our autocorrelation approach thus provides a means to monitor the distribution of fluorescent proteins across the short-axis of the cell during time-lapse measurements: membrane-associated protein produces distinctive cross-peaks, whereas cytosolic/nucleoid associated protein does not.

We then applied autocorrelation analysis to our experimentally acquired UmuC-mKate2 time-lapse images (**Fig 3F**), beginning at the time in which cells were irradiated at the UV dose indicated. Within the time-lapse images, the orientation of *E*. *coli* cells was biased by the flow of medium through the flow cell: the majority of cells aligned with the vertical axis of the images. This orientation bias allowed us to look for the repeating pattern of membrane-localized UmuC-mKate2 signals by measuring image-wide autocorrelation functions across the horizontal axis of the images. The autocorrelation functions were normalized by cellular intensity across the entire dataset of UV doses. At 1 J/m^2^, there was little change in the autocorrelation function during the time-course and little detectable membrane localization. At 3 J/m^2^, a modest signal from membrane-localized UmuC appears late in the time-lapse. At 10 J/m^2^, two distinctive membrane-bound cross peaks appear at about 90 min and persist until 180 min. At 30 J/m^2^ and 100 J/m^2^, membrane localization is prominent from 90 min. From 120–180 min, the autocorrelation is significantly broader, with the valley between the central peak and the membrane peak becoming filled in. This broadening is indicative of UmuC entering the cytosol.

**Fig 4:**
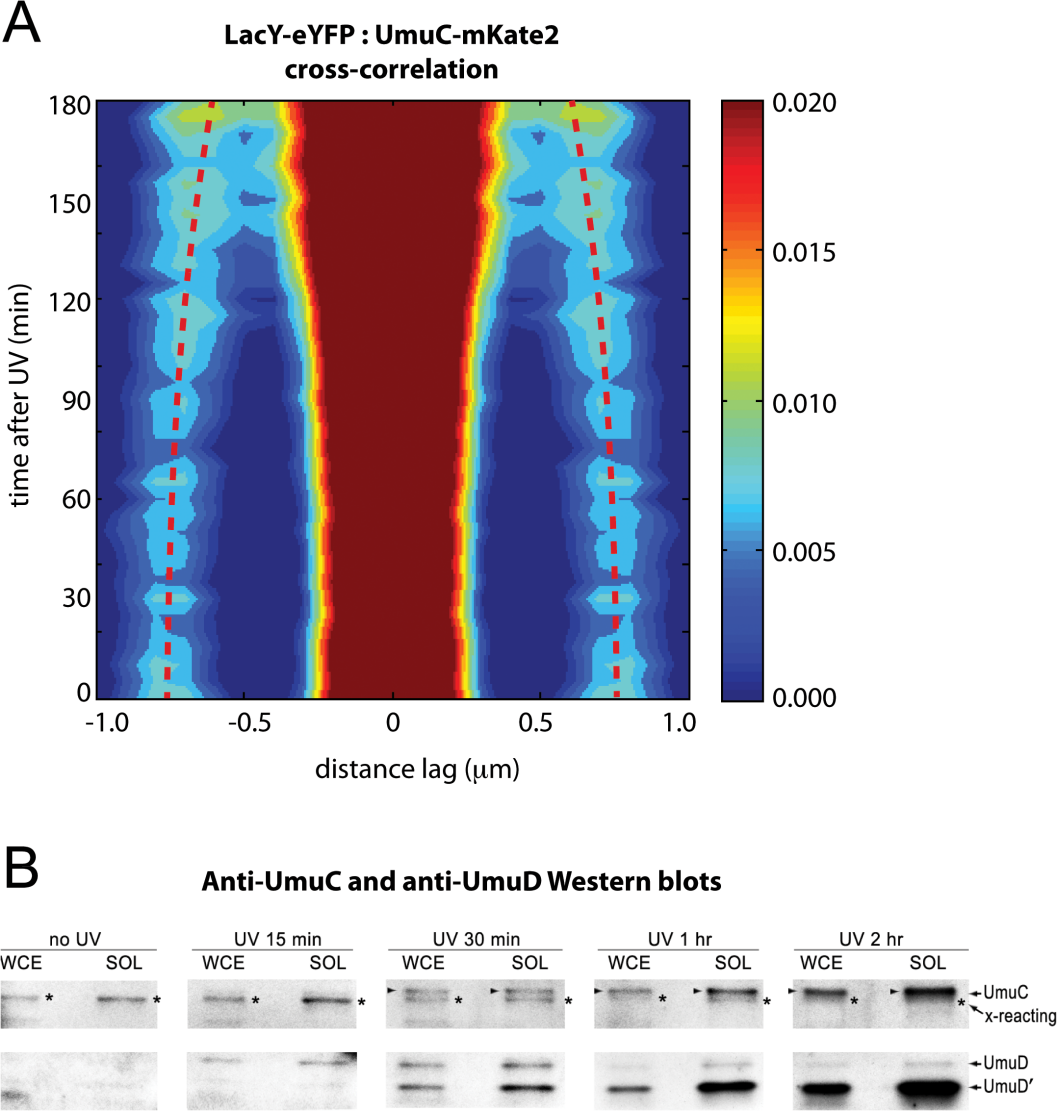
Visualization of UmuC redistribution by cross-correlation analysis and Western blotting. (A) Cross-correlation analysis of LacY-eYFP and UmuC-mKate2 signals from time-lapse measurements (EAW191 containing pBAD-LacY-eYFP). Distance-dependent correlation values between LacY-eYFP and UmuC-mKate2 signals are presented as 2D contour plots. Blue areas indicate low correlation, whereas red areas indicate high correlation. Image pairs containing membrane-associated UmuC-mKate2 foci, thus co-localizing with membrane-defining LacY-eYFP signals, produce sharp cross-correlation peaks at lag = 0 and ± 0.8 μm. Image pairs containing cytosolic UmuC-mKate2 foci produce broader cross-correlation peaks at values between 0 and 0.8 μm. Red dotted lines mark the position of the secondary peaks in time and highlight a time-dependent trend towards lag values < 0.8 μm. A peak-enhancing filter was applied to fluorescence images prior to autocorrelation analysis. (B) UmuD, UmuD' and UmuC expressed from the *recA* ^+^ *lexA* ^+^ strain, RW118, after exposure to 30 Jm^-2^ was monitored in whole cell extracts (WCE) and soluble fraction (SOL) at various time points after irradiation. The protein that is slightly smaller than UmuC and cross-reacts with the UmuC antibodies is indicated with an asterisk to the right of each lane. When detectible, UmuC is indicated by an arrow on the left of each lane. The UmuD and UmuD' proteins are indicated on the right hand side of the image

Evidence of redistribution is also visible in cross-correlation analysis between UmuC-mKate2 and the inner membrane protein LacY-eYFP (**Fig 4A**). We found that expression of the LacY-eYFP protein partially induced the SOS response: cells were longer than usual and produced an elevated level of UmuC-mKate2 prior to UV irradiation. At early time-points after UV irradiation, clear cross-peaks are visible due to co-localization of UmuC-mKate2 and LacY-eYFP on the membrane. Each UmuC-mKate2 focus correlates with LacY-eYFP signal on both the same side of the cell (producing a central peak at lag = 0) and the opposite side of the cell (producing cross-peaks at lag = ± 0.8 μm). From 90–180 min after irradiation, these cross-peaks angle in towards the central peak and become broader. This is consistent with UmuC-mKate2 foci forming in the cytosol, where the distance from each focus to either side of the membrane falls between 0 and 0.8 μm. As UmuC-mKate2 is no longer confined to the membrane, the distance to each side of the membrane becomes more variable, causing the cross-peaks to broaden.

The redistribution of native chromosomally expressed UmuC into the cytosol was also followed by Western blotting of whole-cell extracts (WCE) and soluble fractions at various time points after UV irradiation. (**Fig 4B**). As previously reported, basal levels of UmuD and UmuC in a *recA* ^+^ *lexA* ^+^ strain are undetectable [15]. The affinity purified UmuC antibodies do, however, cross-react with a protein that is slightly smaller than UmuC (**S1A Fig**) that can be detected in both the WCE and soluble fractions (**Fig 4B**). Importantly, the solubility of this cross-reacting protein does not change after UV. We can therefore use this cross-reacting protein as an internal control for the amount of cellular material loaded in each lane. We begin to observe the appearance of soluble UmuC 30 minutes after UV irradiation. The amount of UmuC in the soluble fraction increases further 1 hour and 2 hours post-UV, consistent with the release of UmuC into the cytosol. The extent of soluble UmuC shows an excellent correlation to the amount of soluble UmuD' in the cell. Fifteen minutes post-UV, we observe the induction of UmuD and very little conversion to UmuD'. By 30 minutes post-UV, roughly 50% of the UmuD is converted to UmuD' in the WCE. Much more UmuD' is found in the soluble fraction, indicating that UmuD' is inherently more soluble than UmuD. The amount of UmuD' in the soluble fraction continues to accumulate 1 hour and 2 hours post-UV.

Finally, mapping the cellular location of individual UmuC molecules visualized in cells grown in shaking culture also indicates redistribution of UmuC-mKate2 from the membrane to the cytosol beginning 90 min after UV irradiation (**S7 Fig)**. These results confirm that the membrane localization is observed independent of whether the cells grow in culture or on cover slips, and independent on whether autocorrelation analysis is used or direct mapping.

**Fig 5:**
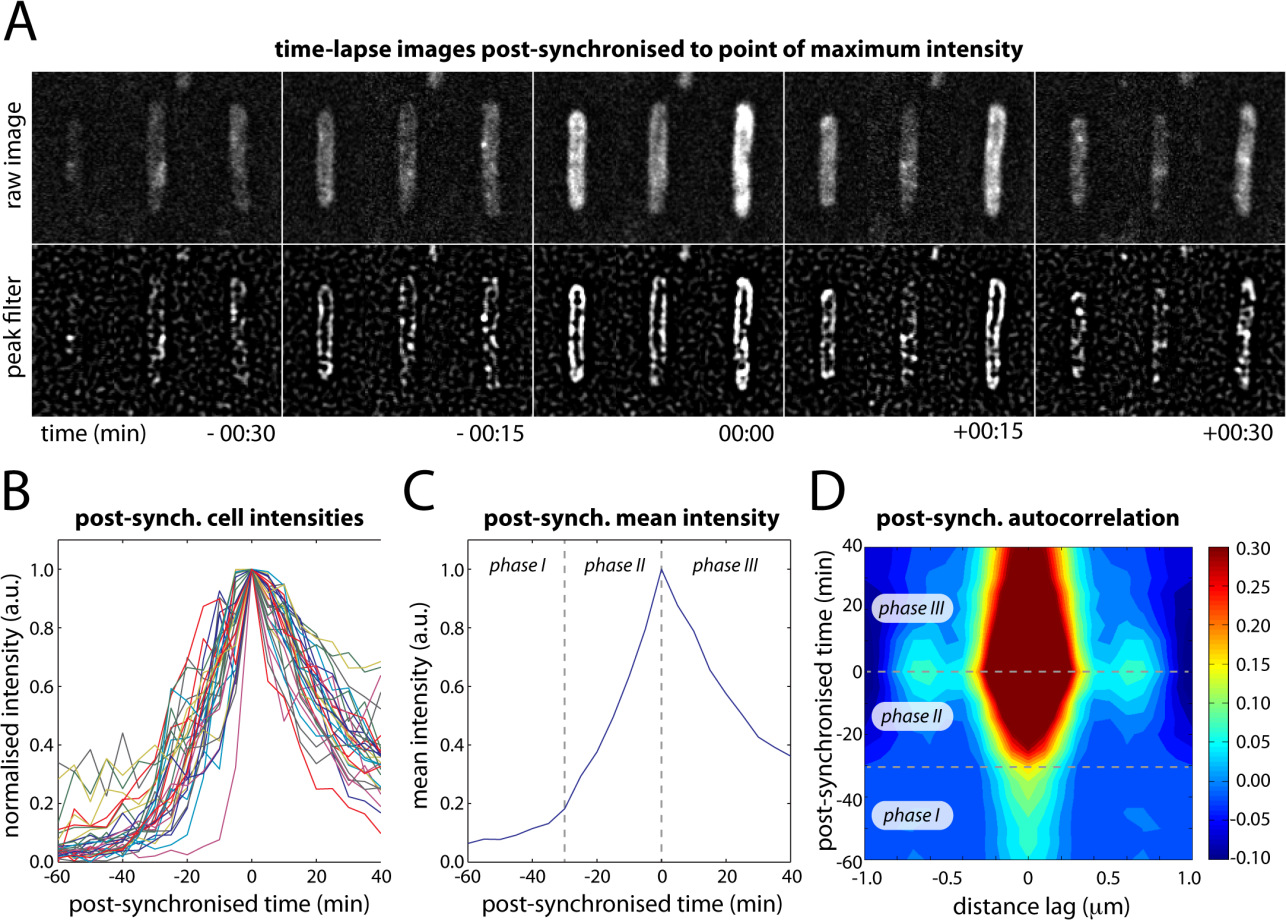
Post-synchronization of individual cells within time-lapse series (EAW282). (A) Time-lapse images of individual cells post-synchronized to the point at which the intensity of UmuC-mKate2 signal reached a maximum (typically 90–150 min after treatment with 100 J/m^2^ UV light). (B) Post-synchronized intensity vs time trajectories for 31 individual cells (of 100 total) that produced a single well-defined burst of UmuC-mKate2 synthesis. The remaining 69 cells either produced no detectable bursts of UmuC-mKate2 synthesis or produced multiple bursts of synthesis, preventing post-synchronization. (C) Mean intensity vs time trajectory over all cells showing three phases in the production of UmuC: (phase I) little UmuC-mKate2 produced; (phase II) increased production of UmuC-mKate2; (phase III) production of UmuC-mKate2 ceases and cellular levels decline. (D) Autocorrelation analysis of post-synchronized time-lapse series showing that the three phases in the production of UmuC correspond with changes in its cellular location: (phase I) weak membrane cross-peaks indicate low levels of membrane-associated UmuC-mKate2; (phase II) stronger membrane cross-peaks indicate production of membrane-associated UmuC-mKate2; (phase III) decreasing membrane cross-peaks and broadening of origin peak indicating redistribution of UmuC-mKate2 to the cytosol. A peak-enhancing filter was applied to fluorescence images prior to autocorrelation analysis [57].

We observed that at large doses of UV light (> 30 J/m^2^), many cells produced a single large burst of UmuC-mKate2 and that the change from membrane-associated to cytosolic distribution seemed to initiate when the level of UmuC reached a maximum. This maximum always occurred more than 90 min after UV. To examine this phenomenon further, we cropped movies of single cells from our time-lapse series (100 J/m^2^) and post-synchronized them each individually to the time point with the maximum fluorescence intensity (**Fig 5A and 5B)**. Where appropriate, the cells were also rotated to align with the y-axis of the image to further enhance the sensitivity of autocorrelation analysis. These post-synchronized movies reveal that during bursts of production (lasting typically 30–60 min) UmuC is membrane-associated and gradually becomes cytosolic after reaching peak intensity (**Fig 5A**).

Autocorrelation analysis on the post-synchronized movies revealed three distinct phases in the behaviour of UmuC (**Fig 5C and 5D**). In phase I, very little UmuC is produced and those molecules that are present associate with the cell membrane. In phase II, substantially more UmuC is produced and these molecules accumulate at the membrane. In phase III, production ceases and UmuC redistributes into the cytosol. The redistribution of UmuC into the cytosol is essentially complete 30 min after the cell reaches peak intensity. The fact that UmuC only redistributes to the cytosol during phase III and after its production has ceased indicates that UmuC is being released from the membrane, as opposed to newly synthesized UmuC becoming sequestered in the cytosol.

### UmuC membrane release requires UmuD cleavage

**Fig 6:**
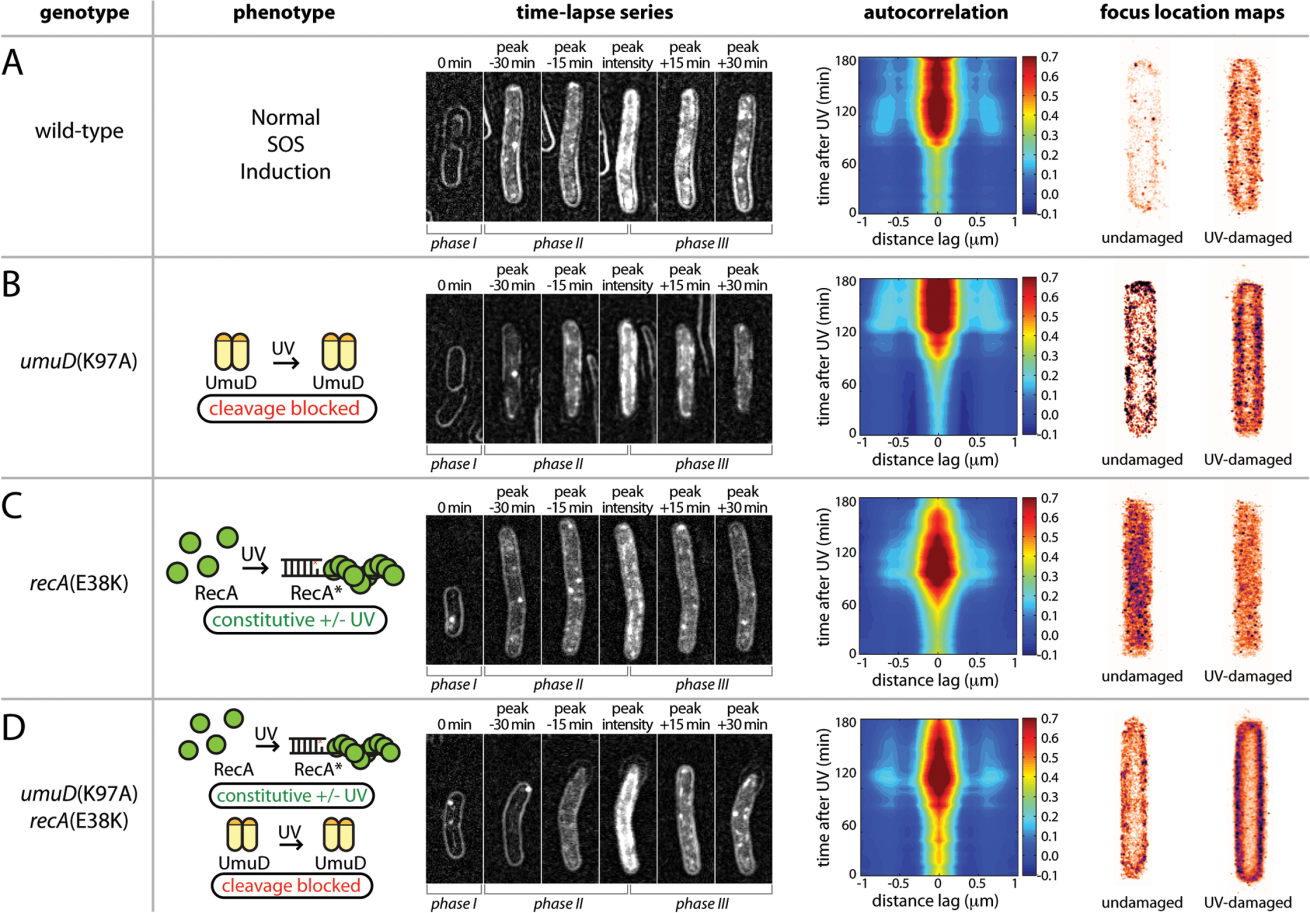
Identifying the cellular location of UmuC-containing species using mutants with defects in the pol V activation pathway. (A-D) Analysis of UmuC-mKate2 localization in strains with different genetic backgrounds; EAW282 (*recA* ^+^ *lexA* ^+^); EAW329 [*recA* ^+^ *lexA* ^+^ *umuD*(K97A)]; EAW307 (*recA*(E38K) *lexA* ^+^); and EAW455 [*recA*(E38K) *lexA* ^+^ *umuD*(K97A)]. For time-lapse/autocorrelation analysis, cells were treated at t = 0 min with UV light at 30 J/m^2^. The images shown in time-lapse series are composites of bright-field images (cell outlines), raw fluorescence images and peak-filtered fluorescence images. The intensity range used for each channel was the same for all composites. The images represent stages in the UV-damage response (left to right: immediately after irradiation; peak signal—30 min; peak signal—15 min; peak signal; peak signal + 15 min; peak signal + 30 min). Autocorrelation analyses are again presented as a 2D contour plots, with the time course beginning at 0 min at the bottom. A peak-enhancing filter was applied to fluorescence images prior to autocorrelation analysis [57]. Side peaks in the autocorrelation function at -0.6 μm and 0.6 μm indicate membrane-associated UmuC-mKate2. More intense cross-peaks indicate a higher proportion of cells with membrane-localised distribution. A broad peak at the origin with no discernable membrane peaks is indicative of cytosolic UmuC-mKate2. Focus location maps (far right panels) were produced from analysis of shaking-culture cells. Cells were grown in EZ medium with glucose at 37°C. The entire 0.5 ml culture was irradiated with 10 J/m^2^ UV light while sandwiched between two quartz plates then returned to shaking culture. Aliquots were taken every 10 min, placed on an APTES-treated coverslip, closed in with a second plain glass coverslip and imaged. 96 × 34 ms frames were recorded using 568 nm excitation light at a power of 1800 W/cm^2^. All UmuC-mKate2 foci from all cells were mapped as Gaussians to a cell of standard size and shape. Maps for UV-irradiated cells include images recorded 10–120 min after irradiation.

The change in abundance of soluble UmuC exhibits a strong correlation to the appearance of UmuD' (**Fig 4B**). To test the hypothesis that the interaction between UmuD' and UmuC allows UmuC to relocate to the cytosol, we employed a *umuD*(K97A) strain that cannot convert UmuD to UmuD′ [12]. Compared with the wild-type background (**Fig 6A**), UmuC-mKate2 in the *umuD*(K97A) strain appeared at about the same time after UV irradiation (**Fig 6B**). However, in this strain the UmuC was more strongly membrane-associated in images and produced stronger and more persistent membrane cross-peaks upon autocorrelation analysis (**Fig 6B**). Note that UmuC is stabilized in the cell when in a complex with UmuD′ [10,16], such that degradation of UmuC by the Lon protease limits the formation of UmuC signals when UmuD′ cannot be formed. This indicated a strong tendency for UmuC-mKate2 to accumulate at the membrane, both before and after UV irradiation. Analysis of *umuD*(K97A) cells grown in shaking culture similarly show a strong bias for UmuC-mKate2 to be localized on the cell membrane (**Fig 6B**). Together, these observations support the hypothesis that conversion of UmuD to UmuD′ is critical for re-localisation of UmuC.

To further test this hypothesis, we determined the location of UmuC-mKate2 in a *recA*(E38K) (also known as *recA730*) background, in which active RecA nucleoprotein filaments (RecA E38K*) are formed constitutively in the absence of DNA damage [17]. Under these conditions, the SOS regulon is partially derepressed and much of the temporal regulation normally imposed on pol V is circumvented: pol V is constitutively formed through UmuD cleavage and activated to pol V Mut. However, the ability of RecA(E38K) to spontaneously form nucleoprotein filaments depends upon the cellular concentration of the RecA mutant [15] and RecA(E38K) can be further activated under various conditions [18]. Indeed, UV treatment results in the apparent induction of UmuC in the *recA*(E38K) *lexA* ^+^ background, where all LexA-regulated proteins are expected to already be expressed in the absence of DNA damage. As expected, and in contrast to the *recA* ^+^ *lexA* ^+^, background (**Figs 3–5 and 6A**), in the *recA*(E38K) *lexA* ^+^ strain UmuC was present at all times after irradiation (**Fig 6C**). The UmuC signal increased over the first 90 min after irradiation, with the brief appearance of a signature for membrane-associated UmuC-mKate2 at the peak of UmuC production (~90 min). This observation suggests that higher levels of UmuC after UV treatment went to the membrane first and then became cytosolic. While *recA*(E38K) cells efficiently cleave UmuD to UmuD' in the absence of DNA damage, UmuD' can *only* be generated from full-length UmuD and it is possible that there is sufficient uncleaved UmuD in these cells to transiently allow binding of UmuC to the membrane. The same cells grown in shaking culture show a general cytosolic distribution of UmuC-mKate2 before and 10–120 min after UV irradiation. These observations again indicate that fully activated pol V Mut is largely cytosolic and that the membrane-localized state corresponds to an intermediate species preceding pol V Mut formation.

To rule out the possibility that the cytosolic localization of UmuC-mKate2 in the *recA*(E38K) strain is due to constitutive production of RecA E38K* as opposed to UmuD cleavage, we then determined the location of UmuC-mKate2 in a *recA*(E38K) *umuD*(K97A) background, in which UmuC and UmuD are produced constitutively but UmuD cannot be cleaved to form UmuD'. As already indicated, overall UmuC levels decline because of Lon-dependent degradation of UmuC in the absence of UmuD′. In this strain, UmuC-mKate2 is seen at the membrane at all time points, increasing after UV induction to a higher and persistent level 90 min after UV induction (**Fig 6D**). Autocorrelation analysis revealed clear membrane cross-peaks throughout the measurement, indicating that in this background UmuC-mKate2 remains associated with the membrane in both the presence and absence of damage. The membrane association in this strain is corroborated by a clean membrane-associated distribution of UmuC-mKate2 both before and 10–120 after UV irradiation seen in the same cells in shaking culture (**Fig 6D**).

**Fig 7:**
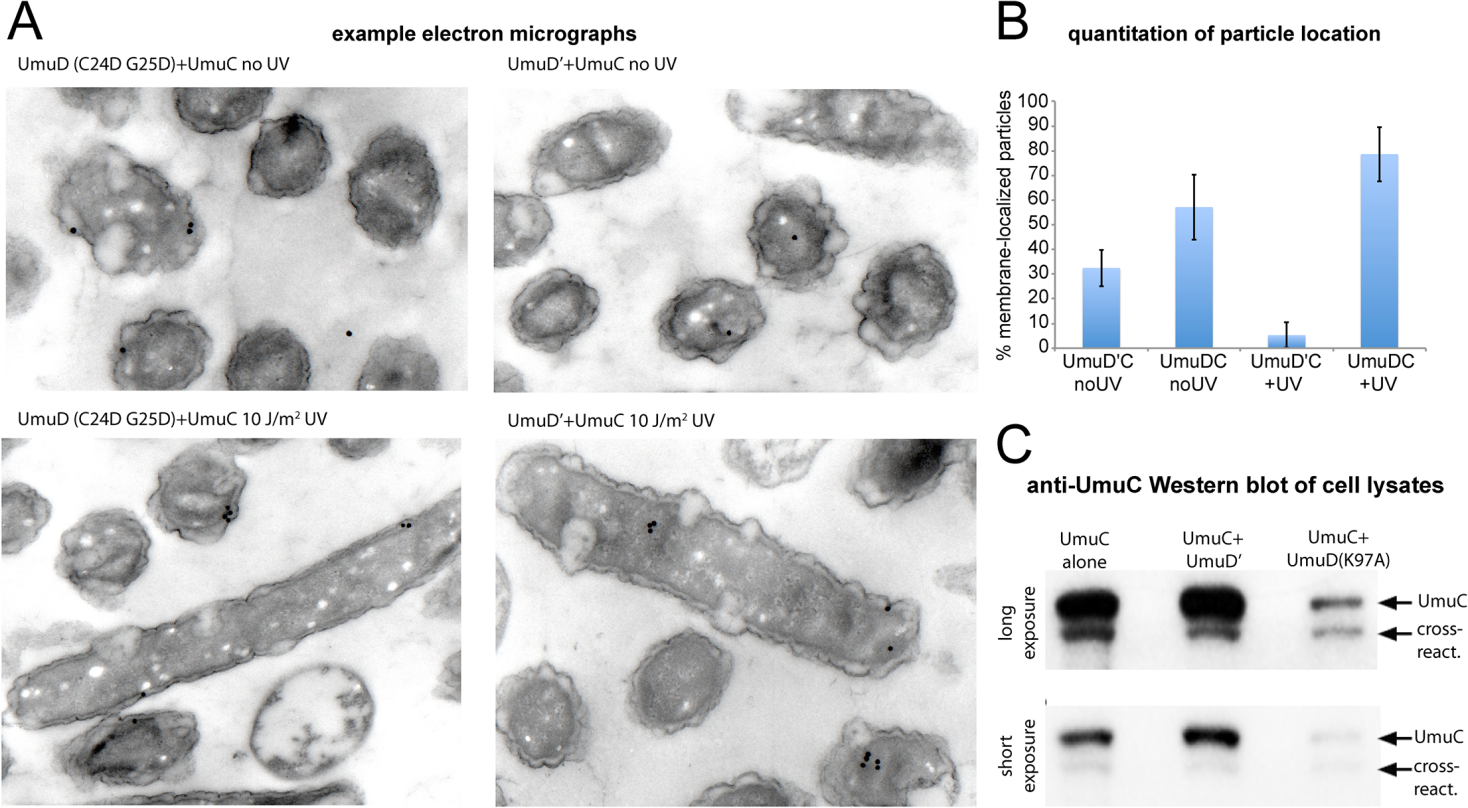
Detection of UmuC location by immuno-electron microscopy and solubility in cell lysates. (A–B) Immuno-electron microscopy of plasmid-expressed UmuC in the presence of *umuD*(C24D G25D) and *umuD*' in the *E*.*coli* strain RW1394. Sectioned electron-microscopy grids were incubated with anti-UmuC antibody and subsequently labeled with 30 nm gold beads conjugated to a secondary goat-anti-rabbit antibody. (A) Representative micrographs. Although the labeling density was low, gold beads were clearly visible for cells expressing UmuC, whereas control cells lacking UmuC showed virtually no beads. (B) Quantification of the proportion of beads associated with the membrane each sample. Beads found to be within 100 nm of the cell membrane were classed as membrane-associated. (C) Levels of soluble UmuC detected by western blotting (*E*. *coli* strain, TCH03). The two images are identical, except that upper panel is a darker exposure of the lower panel. UmuC was expressed alone, or co-expressed with UmuD' or UmuD(K97A). The images clearly show that UmuC is most soluble when co-expressed with UmuD' and least soluble when co-expressed with UmuD(K97A).

The membrane localization of wild-type UmuC when co-expressed with a non-cleavable UmuD protein was also confirmed directly by immuno-electron microscopy (**Fig 7A and 7B)**. Furthermore, in experiments measuring the solubility of wild-type UmuC in *E*. *coli* cell extracts, there appeared to be significantly less soluble UmuC when it was co-expressed with UmuD(K97A) than when expressed alone, or with UmuD' (**Fig 7C**). This is consistent with an active role for UmuD in sequestering UmuC in an insoluble membrane-bound fraction, and/or a requirement for UmuC association with UmuD' to effect release to the cytosol. Together, these results provide clear evidence that conversion of UmuD to UmuD' is a requirement for the spatial relocalization of UmuC to the cytosol.

### Co-localization of pol V mutasomes with replication forks depends on RecA

Having revealed a novel membrane binding and release mechanism underlying the regulation of pol V, we next studied the positioning of pol V molecules after they were released from the membrane, in the context of translesion synthesis. For many years, the prevailing model for pol V-catalysed translesion synthesis was one that described a highly concerted series of events: (i) replisomes stall at lesion sites, exposing ssDNA; (ii) RecA* filaments form on the ssDNA, triggering the SOS response and activating pol V; (iii) pol V displaces pol III through mass action and synthesises over the lesion; (iv) replisomes restart and the cells return to normal. However, a series of recent *in vitro* observations has brought this model into question. First, RecA*-dependent activation of pol V can occur *in trans*, i.e., remote from the site of DNA synthesis [13,19]. Second, replisomes do not necessarily stall at lesions, but can instead skip over them leaving a ssDNA gap in their wake [20]. Finally, the presence of RecA* on the template increases the displacement of pol III by translesion synthesis polymerases, indicating additional complexity above a simple mass action-driven exchange mechanism [21].

Our single-molecule observations allow us to address whether translesion synthesis takes place at replication forks by simultaneously imaging UmuC fused to the red mKate2 and the τ subunit of the DNA pol III holoenzyme fused to the yellow YPet; DnaX-YPet) [22]. We recorded two-color time-lapse series of wild-type *recA* ^+^ cells and constitutively active *recA*(E38K) cells following irradiation with UV light at 30 J/m^2^. We then measured cross-correlation functions between the two image channels as a function of time. This procedure was similar to the autocorrelation-based one described above, except that image color pairs were correlated with each other rather than each image being correlated against itself. Image pairs containing co-localized fluorescent spots should return a strong, sharp cross-correlation signature, whereas spatially unrelated spots should return weak or no cross-correlation.

**Fig 8:**
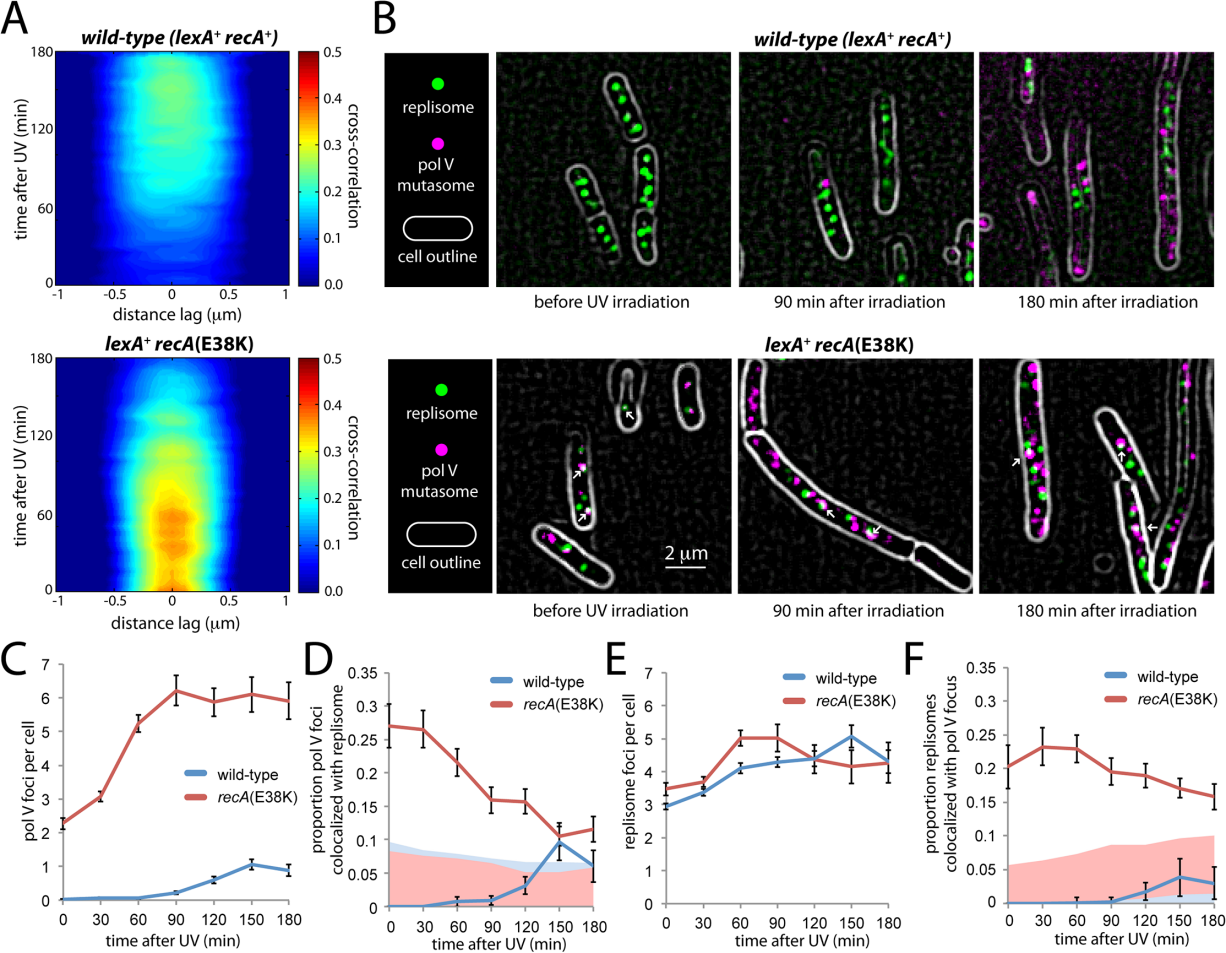
Colocalization of replisomes and mutasomes in wild-type (EAW282) and *recAE38K* cells (EAW307). (A) Cross-correlation analysis of DnaX-YPet and UmuC-mKate2 signals from time-lapse measurements. Distance-dependent correlation between DnaX-YPet and UmuC-mKate2 signals are presented as 2D contour plots. Blue areas indicate low correlation, whereas red areas indicate high correlation. Image pairs containing co-localized replisome and mutasome foci produce high cross-correlation values over a short distance range. A peak-enhancing filter was applied to fluorescence images prior to autocorrelation analysis [57]. (B) Average projections of time-sampling movies showing replisome and mutasome foci. Arrows indicate co-localized replisome-mutasome pairs. A peak-enhancing filter was applied to fluorescence images prior to autocorrelation analysis [57]. (C-F) Changes in replisome and mutasome foci following 30 J/m^2^ UV irradiation. (C) Number of mutasome foci per cell. (D) Proportion of mutasome foci that colocalize with a replisome focus. (E) Number of replisome foci per cell. (F) Proportion of replisome foci that colocalize with a mutasome focus.

In the wild-type background, a broad and relatively weak cross-correlation peak was observed, suggesting little co-localization between DnaX-YPet and UmuC-mKate2 molecules beyond their presence within the same cell (**Fig 8A**). In the *recA*(E38K) background, stronger cross-correlation was observed across a relatively narrow distance regime, consistent with co-localizing spots of diffraction-limited size (**Fig 8A**). However, the cross-correlation declines with time after UV treatment. This observation suggests that in contrast with the wild-type background, a significant proportion of UmuC-mKate2 molecules (primarily in the form of pol V Mut in this background) co-localize with replisomes in the *recA*(E38K) background under normal growth, but that the co-localization declines at later times after UV irradiation.

To probe this co-localization further, we analysed individual cells within the time-sampled movies. Pol V Mut synthesizes DNA at a very low rate (0.3–1 nt·s^-1^) [11]. It can therefore be assumed that pol V Mut molecules forming active mutasomes must remain immobilized on the DNA for at least a few seconds. These molecules should present as static foci in our fluorescence movies (at 34 ms per frame). Making average projections of these movies allowed us to highlight static foci while blurring both transient DNA binders and mobile membrane-associated molecules into the diffuse cellular background **(Fig 8B)**. Foci that persisted for > 300 ms were identified as static foci.

Static foci were observed in UV-irradiated, wild-type *recA* ^+^ cells as well as in both untreated and UV-irradiated *recA*(E38K) cells (**Fig 8B**). In both backgrounds, the number of static foci increases in response to UV-irradiation in a dose-dependent fashion (**Fig 8C**). In the *recA*(E38K) background, a large proportion of static foci co-localize with replisomes in the absence of UV (**Fig 8D**). These observations strongly support the notion that the static foci we observe represent DNA-bound pol V Mut molecules. From this point forward we will refer to these static foci as mutasome foci.

In the wild-type background, mutasome foci appear in the cytosol 90 min after damage was induced (**Fig 8B and 8C**), 30 min after the point where UmuC levels begin to increase (**Fig 2**). On average, cells contained 1.1 ± 0.2 mutasome foci at the peak time of 150 min. Very few of these co-localized with replisomes: the percentage detected as being co-localized fell within the range expected by chance (based on the area of the cell occupied by replisome foci, ~5–10%, **Fig 8D**). These observations are consistent with the weak cross-correlation signature we observed in the time-lapse analysis (**Fig 8A**).

In the *recA*(E38K) strain, pol V Mut is produced constitutively and is evenly distributed throughout the cytosol both before and after inducing damage (**Fig 6B, S8 Fig**). Prior to UV irradiation, static foci are observed in the cytosol, averaging 2.3 ± 0.2 per cell (**Fig 8B and 8C**). In contrast to the behavior observed in wild-type cells, a significant proportion of these foci (27 ± 3%, n = 111 cells) co-localize with replisomes (**Fig 8D**). This observation is consistent with the strong cross-correlation observed between DnaX-YPet and UmuC-mKate2 in the corresponding time-lapse measurements (**Fig 8A**) and indicates that pol V Mut gains access to replisomes under these conditions. This observation is consistent with the elevated rate of mutagenesis (~ 100-fold) observed in the *recA*(E38K) (*recA730*) background in the absence of damage [17]. As seen in the cross-correlation (**Fig 8A**), we observe that after UV irradiation, the co-localization with replisomes gradually decreases (**Fig 8D**). By 90 min after UV, relatively few pol V Mut foci colocalize with replisomes (16 ± 2%, n = 84) and by 150 min co-localization (11 ± 1%, n = 90) approaches levels expected to occur by chance (~5–10%, **Fig 8D**). The proportion of replisomes that co-localized with a static pol V focus remained approximately the same throughout the measurement, indicating that pol V Mut gains similar levels of access to replisomes in both the presence and absence of damage in the *recA*(E38K) background (**Fig 8E and 8F**). Analysis of cells grown in shaking culture returned similar results: there is no significant co-localization of mutasomes and replisomes in the wild-type background; a significant proportion of mutasomes do co-localize with replisomes in undamaged *recA*(E38K) cells; this co-localization gradually diminishes after UV irradiation (**S9 Fig**).

Overall, our results show that mutasomes form at replisomes in undamaged *recA*(E38K) cells in which the SOS response is constitutive, but few sites for DNA synthesis exist outside of replisomes. After damage, additional mutasomes are assembled at sites that are spatially distinct from replication forks. In the wild-type background, static foci rarely form at replication forks, clearly questioning the canonical view of mass-action driven binding of pol V at stalled replication forks.

## Discussion

**Fig 9:**
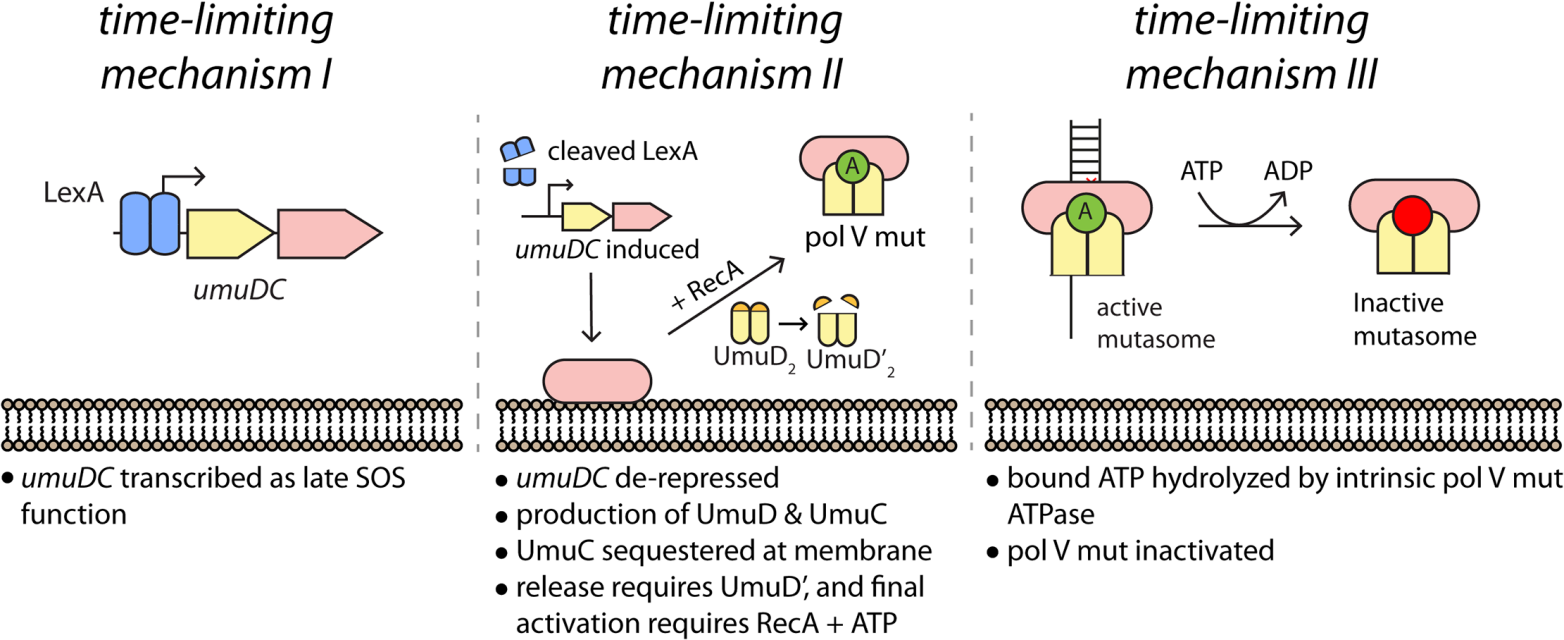
Multiple mechanisms limit pol V activity on DNA. (I) The activity of low fidelity pol V is regulated at the transcriptional level, with gene transcription and translation delayed by tight binding to the *umuDC* operator by LexA protein. (II) As documented here, activation of pol V is further delayed by sequestration of UmuC (and possibly UmuD) in the membrane. Release and final activation requires the conversion of UmuD to UmuD'. (III) Once activated, the length of time that pol V Mut is operative in TLS DNA replication is dependent on an ATPase activity intrinsic to the enzyme [14]. The symbol “A” represents a molecule of ATP bound to RecA in the activated pol V Mut complex.

This work reveals a new level of regulation in the activation of DNA polymerase V. Previous work has defined two molecular mechanisms that limit the amount of time that pol V has to access DNA templates. The first is the transcriptional, posttranslational and proteolytic regulation of pol V, ensuring that the polymerase accumulates relatively late in the SOS response [4,9]. The second consists of an intrinsic ATPase activity that limits the number of nucleotides incorporated by pol V Mut once TLS commences [14]. The work described here reveals that the UmuC and UmuD proteins are not activated as soon as they appear. Instead, activation is further delayed by sequestration of UmuC (and probably UmuD as well) at the inner cell membrane. The resulting delay constitutes another time-limiting mechanism that functions between the other two (**Fig 9**). This new layer of regulation is not merely temporal, but also introduces a novel spatial dimension to the system. Release from the membrane requires the conversion of UmuD to UmuD'. These many layers of control help ensure that pol V activation is limited to circumstances where the extent of damage exceeds the cell’s capacity for error-free repair. Once activated, many pol V Mut foci do not co-localize with DNA pol III replisomes, suggesting that pol V Mut may often function at lesion-containing gaps left behind by the replicative polymerase.

Time-lapse analyses revealed three distinct phases in the regulation of pol V activity within UV-irradiated cells. In phase I, little UmuC is produced. The few molecules that are present at this stage are primarily associated with the cell membrane. In phase II, the number of UmuC molecules in the cell increases sharply as the *umuDC* operon is derepressed by cleavage of LexA. Presumably, the majority of other SOS-regulated proteins are also induced during this phase. During phase II, UmuC associates with the cell membrane. In phase III, UmuC is no longer expressed and its cellular levels decrease. During this phase, UmuC gradually enters the cytosol as pol V Mut, forming mutasomes on the DNA at sites spatially distinct from replisomes. The transition from phase II to III requires UmuD cleavage to UmuD'. This represents not only a biochemical switch that allows formation of active pol V Mut, but also a spatial switch that releases UmuC from the membrane.

Spatial organization is a common feature of regulation in eukaryotes, where many processes are tied together by protein scaffolds or segregated into compartments [23–32]. Well-characterized examples are much less common in bacteria. The transient sequestration of UmuC in the bacterial inner membrane provides the first example of spatial regulation for a bacterial DNA processing enzyme. It is interesting to speculate that the inner membrane may be a more general repository of DNA metabolism enzymes that are temporarily not needed but still readily available if required.

Our unexpected observation of prescribed changes in the spatial distribution of UmuC with time after exposure to UV raises new questions about the mechanisms of translesion synthesis in *E*. *coli*. The results show that *in vivo*, UmuC interacts with the cell membrane and is released as soluble pol V following RecA*-mediated conversion of UmuD_2_ to UmuD′_2_. How does UmuC become associated with the membrane? When expressed alone, the vast majority of UmuC is naturally insoluble, yet a small fraction can nevertheless be recovered in soluble cell extracts (**Fig 7C**). The amount of soluble UmuC increases when co-expressed with UmuD′ (**Fig 7C**) [11,33], suggesting that the insoluble UmuC is not mis-folded, but rather is simply too hydrophobic to enter aqueous solution by itself. It would therefore be reasonable to hypothesize that such hydrophobicity might promote a direct interaction of UmuC with the cell membrane. Our measurements, however, indicate that other factors are involved. For example, the amount of soluble UmuC decreases significantly when co-expressed with non-cleavable UmuD(K97A) (**Fig 7C**). This observation suggests that full-length UmuD also plays a role in helping to sequester UmuC in an insoluble form, (most likely on the inner membrane), an issue that is under further investigation.

It is possible that other DNA damage-induced proteins might also be involved in sequestering UmuC at the membrane. Thirty years ago, evidence was published for DNA damage-dependent association of RecA with the cell membrane [34]. Both UmuC and UmuD are known to interact with RecA [35–37], thus it would be interesting to determine if membrane-localized RecA also plays a role in sequestering UmuC. Because of the many roles that RecA plays in the regulation of pol V, however, examining this possibility would be extremely challenging experimentally and is beyond the scope of the current study.

How might membrane association provide control over mutagenic pol V activity? At the simplest level, sequestration of the bulk of UmuC at the membrane would leave few pol V Mut molecules free in the cytosol with access to DNA. A delay between production of UmuC and formation of cytosolic pol V Mut provides an expanded window of time in which error-free DNA repair proteins (which have weaker affinity LexA-binding sites and thus are highly unlikely to be co-expressed with UmuC) can repair the damaged DNA before mutagenic pol V lesion bypass takes place. Ideally, one would directly examine the links between membrane-sequestration of UmuC and pol V-dependent mutagenesis rates, independently of biochemical switches that regulate pol V activity. Our results show, however, that at least one of these biochemical switches, the conversion of UmuD to UmuD', is intimately tied to changes in UmuC localization. Currently, no mutants exist that would allow for separation of these functions. What is clear, however, is that the *umuD*(K97A) mutation, which prevents release of pol V from the membrane, strongly inhibits pol V-induced mutagenesis [12].

In UV-irradiated wild-type *recA* ^*+*^ cells, pol V mutasomes rarely co-localize with DnaX-YPet at replisomes. Co-localization is frequently observed for *recA*(E38K) cells exhibiting constitutive RecA* activity, but decreases after UV treatment. Previous models for TLS have posited that at stalled replisomes, TLS polymerases displace pol III core (αεθ) from the β sliding clamp, while the rest of replisome, including the clamp loader complex, remains associated [38]. Our observations suggest that for pol V Mut, such an ‘alternative replisome’ mechanism is the exception rather than the rule.

Recent elucidation of pol V Mut as a mobile mutasome complex when first activated [13, 39] provides a facile mechanism for TLS-mediated repair of gaps that are not near the replisome. The fact that UmuC co-localizes with replisomes in an undamaged *recA*(E38K) strain is in excellent agreement with the suggestion that the pol V-dependent spontaneous mutator phenotype of *recA*(E38K) strains is due to a competition between pol III and pol V at a nascent replication fork [40], with most pol V mutator activity occurring on the lagging strand [41]. In an irradiated cell, pol III motion is blocked by the presence of a UV-induced photoproduct, resulting in the replisome dissociating and re-initiating synthesis downstream of the lesion [20,42]. Such a mechanism would leave a lesion-containing single-stranded gap in the chromosome, complete with a β_2_ sliding clamp left behind by the departing replisome. Translesion DNA synthesis in such a gap may represent a major function of pol V Mut.

### Experimental procedures

A complete list of the *Escherichia coli* strains used in this study appears in Table 1.

**Table 1:** Strains used in this study.

Strain	Relevant Genotype	Parent strain	Source/technique
MG1655	*umuC* ^*+*^ *recA* ^*+*^ *lexA* ^*+*^	-	[52]
EAW191	*umuC-mKate2 recA* ^*+*^ *lexA* ^*+*^	MG1655	Lambda RED recombination
RW118	*recA* ^*+*^ *lexA* ^*+*^		[43]
RW120	*recA* ^*+*^ *lexA* ^*+*^ *ΔumuDC*	RW118	[43]
RW1480	*recA* ^*+*^ *lexA* ^*+*^ *umuD* ^*+*^ *umuC-mKate2*	RW120	Transduction of RW120 with P1 grown on EAW191
RW574	*sulA* ^-^ *recA*(E38K) *lexA51*(Def)	-	[44]
RW578	*sulA* ^-^ *recA*(E38K) *lexA51*(Def) Δ*umuDC*	-	[44]
RW1314	*sulA* ^-^ *recA*(E38K) *lexA51*(Def) *umuD* ^+^ *umuC*-*mKate2*	RW578	Transduction of RW578 with P1 grown on EAW191
RW644	Δ*polB*::*Spc ΔdinB*::*Zeo ΔumuDC*::Erm	BL21(λDE3)	Novagen
JW0059	Δ*polB770*::Kan		*E*. *coli* genetic stock center
TCH03	Δ*polB770*::Kan Δ*dinB*::*Zeo ΔumuDC*::Erm	RW644	Transduction of RW644 with P1 grown on JM0059
SG22094	Δ*rcsA*::Kan	-	Susan Gottesman
RW1392	*sulA* ^*-*^ *recA*(E38K) *lexA*51(Def) *ΔumuDC ΔrcsA*::Kan	RW578	Transduction of RW578 with P1 grown on SG22094
SG12047	Δ*lon*::Tet	-	Susan Gottesman
RW1394	Δ*sulA* ^*-*^ *recA*(E38K) *lexA51*(Def) *ΔumuDC ΔrcsA*::Kan *Δlon*::Tet	RW1392	Transduction of RW1392 with P1 grown on SG12047
JJC5945	*dnaX-YPet recA* ^*+*^ *lexA* ^*+*^	MG1655	Benedict Michel
EAW282	*dnaX-YPet umuC-mKate2 recA* ^*+*^ *lexA* ^*+*^	JJC5945	Transduction of JJC5945 with P1 grown on EAW191
EAW307	*sulA* ^-^ *umuC*-*mKate2 recA*(E38K) *dnaX*-*YPet*	EAW288	Transduction of EAW288 with P1 grown on JJC5945
EAW288	*umuC*-*mKate2 sulA* ^*-*^ *recA*(E38K)	EAW297	Transduction of EAW297 with P1 grown on EAW287
EAW297	*umuC*-*mKate2 sulA* ^-^	EAW191	Transduction of EAW191 with P1 grown on EAW13
EAW13	*sulA* ^-^	MG1655	Lambda RED recombination
EAW287	*sulA* ^-^ *recA*(E38K)	EAW16	Lambda RED recombination
EAW16	*sulA* ^-^ Δ*recA*	EAW13	Lambda RED recombination
EAW309	*sulA* ^-^ *umuC*-*mKate2 lexA*(Def)	EAW297	Transduction of EAW297 with P1 grown on EAW26
EAW26	*sulA* ^-^ *lexA*(Def)	EAW13	Lambda RED recombination
EAW329	*umuD*(K97A) *umuC*-*mKate2*	EAW191	Lambda RED recombination
EAW423	*umuD*(K97A) *umuC*-*mKate2 sulA* ^-^	EAW329	Transduction of EAW329 with P1 grown on EAW13
EAW455	*umuD*(K97A) *umuC*-*mKate2 recA*(E38K) *sulA* ^-^	EAW423	Transduction of EAW423 with P1 grown on EAW287

#### Strain and plasmids for mutagenesis assays

The *E*. *coli* K-12 strain RW118 [full genotype:*thr-1 araD139* Δ(*gpt-proA*)*62 lacY1 tsx-33 supE44 galK2 hisG4 rpsL31 xyl-5 mtl-1 argE3 thi-1 sulA211*] and the isogenic *ΔumuDC595*::*cat* strain, RW120, have been described previously [43]. An isogenic strain, RW1480, expressing *umuC*-mKate2:: Kan was made by P1 transduction of the allele from EAW191 into RW120 by selecting for kanamycin resistance and screening for chloramphenicol sensitivity. RW574 and RW578 are isogenic *recA*(E38K) *lexA51*(Def) derivatives of RW118 and RW120 respectively [44]. An isogenic *recA*(E38K) *lexA51*(Def) strain, RW1314, expressing *umuC*-mKate2:: Kan was made by P1 transduction of the allele from EAW191 into RW578 by selecting for kanamycin resistance and screening for chloramphenicol sensitivity.

#### Spontaneous and chemically induced mutagenesis assays

Bacterial cultures were grown overnight in LB medium containing the appropriate antibiotics. Aliquots (1 ml) were centrifuged and resuspended in an equal volume of SM buffer [45]. The ability of each strain to promote Umu-dependent chemically induced mutagenesis was judged by plating 100 μl aliquots on minimal agar plates supplemented with a trace amount of histidine (1 μg/ml) [46]. Immediately after plating, 5 μl of a 1:5 dilution of methyl methane sulfonate (MMS, Sigma) in dimethyl sulfoxide (DMSO, Sigma) was applied to a small sterile disk in the center of the plate. His^+^ revertants were scored after 4 days of incubation at 37°C. The number of His^+^ revertants is expressed as the mean number of MMS-induced mutants per plate after subtracting the mean number of spontaneously arising His^+^ revertants on plates that were not exposed to MMS and were obtained from 5 individual cultures plated in triplicate for each assay.

#### Quantitative UV-induced mutagenesis assays

Spontaneous and damage induced reversion of the *hisG4* locus was assayed as previously described [44]. Cells were grown overnight at 37°C in LB media. The overnight culture was diluted 1:100 in fresh LB media and grown until an OD_600_ ~0.4. Cells were harvested by centrifugation and resuspended in an equal volume of SM buffer [45]. Cells were irradiated with UV-light (254nm) in a petri dish at a UV-fluence of 0.5 J/m^2^ per second. Aliquots were removed at each UV dose and serial dilutions of the culture spread in triplicate on minimal agar plates supplemented with a trace amount of histidine (1 μg/ml). On these plates, less than 1000 viable bacteria grew on the low level of histidine to form small detectable colonies after 2 days. When ~ 4x10^7^ bacteria were seeded, they grew to form a lawn, concomitantly exhausting the low level of histidine. His^+^ mutants grew up through the lawn and were counted after 4 days. The number of pre-existing spontaneous mutants was determined by seeding on minimal plates lacking histidine. The UV-induced mutation frequencies were calculated as previously described [47] and takes into account spontaneously arising mutants, as well as any cell killing that reduces the number of pre-existing His^+^ mutants in the culture. Experiments were carried out at least three times and were performed under yellow light to avoid unwanted photoreactivation.

#### Strains for expression and solubility assays

The expression strain, RW644 (BL21(λDE3) Δ*polB*::Spec Δ*dinB*::Zeo Δ*umuDC*::Erm) has been described previously [11]. TCH03 is isogenic with RW644 and was made by transducing Δ*polB770*::Kan from JW0059 (*E*. *coli* Genetic Stock Center) into RW644 selecting for kanamycin resistance and screening for sensitivity to spectinomycin. The wild-type *umuC* gene was chemically synthesized by Genscript (Piscatawy, NJ) and cloned into pET22b+ as an ~1.3kb NdeI-XhoI fragment, to generate pJM1113. A plasmid expressing mKate2-UmuC was generated by the chemical synthesis of a DNA fragment containing the 3' end of *umuC* in frame with the *mKate2* gene (Genscript), and was cloned into the BamHI-XhoI sites of pJM1113 to generate pJM1115. pJM1116 and pJM1117 are low-copy spectinomycin resistant plasmids that constitutively express UmuD' or UmuD(K97A) from the RecA(Oc) promoter. They were constructed by subcloning the *umuD*' gene from pJM105 [48] as an NdeI-PstI fragment into pJM1071 and the *umuD*(K97A) gene as an NdeI-BglII fragment from pEC69 [49] into NdeI-BamHI digested pJM1071, respectively.

#### Western blotting for UmuC expression and solubility

Intracellular levels of UmuD' and UmuC proteins was determined by western blotting using affinity purified polyclonal UmuD' and UmuC antisera using the protocols previously described [15]. Overnight cell cultures were diluted 1:100 in fresh media and grown at 37°C until they reached an OD_600_ ~0.5. Cultures were centrifuged and resuspended in 8 ml of Buffer A (50mM Tris pH 7.5, 300 mM NaCl and 10% glycerol). Cells were lysed by sonication on ice for a total processing time of 3 min. The suspension was then centrifuged at 35,000 RPM for 30 minutes and the supernatant saved as the soluble cell extract. Protein extracts were separated on a NuPAGE 4–12% Bis-Tris gel and transferred to a Novex PVDF membrane. The membrane was incubated overnight with a 1:1000 dilution of affinity purified polyclonal rabbit antisera raised against UmuC. The next day, it was washed twice and subsequently incubated for 90 minutes with a 1:10,000 dilution of goat anti-rabbit IgG conjugated to alkaline phosphatase (Applied Biosystems; cat # T2191). Finally, the membrane was treated with a 1:50 dilution of Tropix CDP-Star substrate (Applied Biosystems) in 1X assay buffer for 10 min. Proteins were visualized by exposing the membrane to X-ray film for various time periods.

#### Strain and plasmids for electron microscopy assays

Strain RW1394 is isogenic with RW578 but also carries the Δ*rcsA*::Kan and Δ*lon*::Tet alleles that were sequentially transduced from SG22094 and SG21047 respectively into RW578. pRW134 expressing UmuD'C and pJM155 expressing a non-cleavable cleavage site mutant of UmuD together with UmuC, are derivatives of the low-copy number spectinomycin resistant vector, pGB2 [50] and have been described previously [49,51].

#### Strain and plasmids for fluorescence assays

EAW282 is *E*. *coli* MG1655 [52] *dnaX*-YPet, *umuC*-*mKate2*. It was made by P1 transduction of JJC5945 to *umuC*-*mKate2* with P1 grown on EAW191. JJC5945 is MG1655 *dnaX*-YPet and was obtained from Benedicte Michel. EAW191 was made by replacing the MG1655 *umuC* gene in its native chromosome location with a *umuC* gene with a C-terminal 11 amino acid spacer followed by mKate2 and a mutant FRT-Kanamycin resistance-wt FRT cassette, via λ RED recombination [53]. The sequence encoding the 11 amino acid spacer (TCCGCTGGCTCCGCTGCTGGTTCTGGCGAGTTC) is that used by Rodrigo Reyes-Lamothe [22] with changes for better codon use and the *EcoRI* site removed. EAW307 is *E*. *coli* MG1655 *sulA* ^–^, *umuC*-mKate2, *recA*(E38K), *dnaX*-*YPet*. EAW191 was transduced to *sulA* ^−^ with P1 grown on EAW13. EAW13 is MG1655 with a FRT-Kanamycin resistance-FRT cassette replacing the *sulA* gene between the SphI and HpaI sites.

The *sulA* ^−^ *umuC-mKate2* strain, designated EAW297, was P1 transduced to *recA*(E38K) with P1 grown on EAW287. EAW287 is MG1655 *sulA* ^–^, *recA*(E38K) with a mutant FRT-Kanamycin resistance-wt FRT located after the C-terminus of the downstream *recX* gene. MG1655 *sulA* ^–^, *umuC-mKate2*, *recA*(E38K) *was* transduced to *dnaX*-*YPet* with P1 grown on JJC5945. EAW309 is *E*. *coli* MG1655 *umuC*-*mKate2 sulA*- *lexA*(Def). It was made by transducing EAW297 with P1 grown on EAW26. EAW26 was made by lambda RED recombination of EAW13 to replace the wild-type *lexA* gene with a deficient *lexA* that had the gene segment between the PshAI and AflIII sites replaced by the Kan^R^ gene. EAW329 is MG1655 *umuD*(K97A) *umuC*-*mKate2* and was made by replacing the *umuDC* genes with *umuD*(K97A) *umuC*-*mKate2* by lambda RED recombination. Both have the same arrangement of *umuC-spacer-mKate2-mutant FRT-KanR-wt FRT* as EAW191.

The plasmid pBAD-LacY-eYFP was a generous gift from J. T. Mika (University of Groningen). It was constructed by inserting *lacY-eYFP* (encoding the lactose transporter LacY fused to the N-terminus of eYFP) into the plasmid pBAD/His (Invitrogen) and allows for L-arabinose-inducible expression of LacY-eYFP. The plasmid pBAD-mKate2 was constructed by inserting *mKate*2 (including an 11 amino acid linker at its N-terminus, sequence TCCGCTGGCTCCGCTGCTGGTTCT-GGCGAGTTC) into pBAD/His (Invitrogen). The linker-mKate2 fragment was created by PCR amplifying (KOD polymerase, Novagen) the mKate2 gene (Evrogen) using forward primer containing the linker sequence (SAGSAAGEF; ATCCGAGCTCGAG ATG TCG GCT GGC TCC GCT GCT GGT TCT GGC GAA TTC ATG GTG AGC GAG CTG ATT AAG GAG) and reverse primer (GTT CCT ATT CTC TAG AAA CTA TAG GAA CTT CTC ATC TGT GC). The amplified fragment and pBAD-myc-hisB were digested with XhoI and XbaI (New England Biolabs) and ligated together with T4 DNA ligase (New England Biolabs, 16°C, overnight) followed by transformation into *E coli* DH5α. Plasmid DNA was isolated from bacterial colonies containing the pBAD-myc-hisB with the ligated linker-mKate2 construct and sequenced to verify the sequence of the linker (GATC biotech).

#### Electron microscopy

*E*. *coli* strain RW1394 (relevant genotype; *recA*(E38K) *lexA51*(Def) Δ*umuDC* Δ*rcsA Δlon*) harboring low-copy-number spectinomycin resistant plasmids pGB2 (vector) [50], pRW134 (recombinant UmuD'C) [51], or pJM155 (non-cleavable cleavage site UmuD mutant, UmuD(C24D-G25D)/UmuC) [49] were grown overnight at 37°C in Davis and Mingioli (DM) minimal media. The overnight culture was diluted 1:100 into 25ml fresh DM media and grown approximately 4 h at 37°C until an OD_600_ ~0.4–0.6. 10 ml of the culture was transferred to a sterile petri-dish and irradiated with 10 J/m^2^ UV-light (254nm) at a fluence of 0.5 Jm^-2^/sec and then returned to a new culture flask. Both unirradiated and irradiated cultures were incubated at 37°C for a further 45 minutes before 1 ml of the culture was transferred to a microfuge tube and centrifuged at ~14,000 x g for 5 mins. The supernatant was decanted and the pellet resuspended in an equal volume of PBS containing 0.5% glutaraldehyde. The suspension was kept on ice for 25 mins before centrifugation and washed 2x with an equal volume of PBS. Cells were embedded in resin and sectioned by Jan Endlich (JFE Enterprises, Beltsville, MD) under a custom service contract. This process entailed lightly centrifuging the cells into a pellet, which were dehydrated in a series of ethanol solutions, (30% EtOH, 50% EtOH and 70% EtOH). Cells were then infiltrated 1:1 with LR White to 70% EtOH, 1:2 LR White to 70% EtOH, 1:3 LR White to 70% EtOH and 3 changes of pure resin. The infiltrated samples were then transferred in gelatin capsules with fresh LR White and then cured for 48 hours at 50°C. Blocks were removed and gelatin capsule sections were cut approximately 60–80nm thin. Sectioned EM grids were returned to us and were placed on 50 μl droplets of sterile PBS containing 0.5% BSA and 0.1% gelatin (PBSBG) for 3 hours at room temperature to reduce any non-specific binding of the UmuC antibodies. After which time, the grids were placed on a 25 μl droplet of a 1:20 dilution of affinity purified polyclonal rabbit antisera diluted in PBSBG and incubated overnight at 4°C. Grids were washed 3x by placing on sequential drops of 50 μl PBSG for 10 mins each. Grids were then placed on a 25 μl droplet of a 1:40 dilution of goat-anti-rabbit antibody (in PBSBG) conjugated to 30nm gold particles (Abcam, cat # ab119178) and incubated overnight at 4°C. The grids were washed 3x with PBSG for 10 mins each and dried by blotting on filter paper. Grids were then returned to Jan Endlich (JFE enterprises, Beltsville, MD), who stained them for 5 minutes with 2% aqueous uranyl acetate and then examined and imaged the cells with a Ziess EM 10C transmission electron microscope.

#### Fluorescence microscopy

We constructed a wide-field single-molecule fluorescence microscope by coupling high power laser excitation into a commercially available inverted fluorescence microscope body (IX-81, Olympus) equipped with a 1.49 NA 100x objective and a 512 × 512 pixel EM-CCD camera (C9100-13, Hamamatsu). Excitation light was provided continuous-wave optically pumped semidiode lasers (Sapphire LP, Coherent) of wavelength 514 nm (150 mW max. output) and 568 nm (200 mW max. output). For imaging UmuC-mKate2 fusions we used 568 nm excitation light at high intensity (180–1800 W.cm^-2^) and collected light emitted between 610–680 nm (ET 645/75m filter, Chroma). For imaging DnaX-YPet we used 514 nm laser excitation at lower power (10 W.cm^-2^) and collected between 525–555 nm (ET540/30m filter, Chroma). For LacY-eYFP imaging we used 514 nm excitation at high power (1800 W.cm^-2^). Image analysis was performed with ImageJ [54].

For imaging we used glass coverslips derivatized with 3-aminopropyl triethoxy silane (APTES, Sigma). Coverslips were first cleaned by sonicating for 60 min in 5M KOH and then thoroughly washing with water. The coverslips were then treated for 2 h with 2% APTES in water before being washed thoroughly with water, then acetone and dried before assembling flow cells. Our flow cell design was that used in reference [55]. Typical channel dimensions were 3 × 30 × 0.03 mm (length × width × height).

#### UV irradiation and imaging

For most imaging experiments, cells were grown at 37°C in EZ rich defined medium (Teknova) that included 0.2% (w/v) glucose. Cells containing pBAD-LacY-eYFP were grown in EZ that contained 0.2%(v/v) glycerol and 0.001% (w/v) L-arabinose. For time-lapse and time-sampling imaging we mounted APTES flow cells to our microscope, maintaining the temperature at 37°C by a combination of stage heating and objective lens heating. Cells were grown in shaking culture until reaching mid-log phase (OD_600_ ~ 0.5) before being loaded into flow cells by pulling with a syringe. The inlet was then placed into fresh medium that was constantly aerated using an aquarium pump. Medium was then pulled through the flow cell using a syringe pump, at a rate of 30 μl/min. Cells were irradiated *in situ* with 254 nm UV light from a mercury lamp (UVP) at a fluence of 1–100 J.m^-2^.

#### Super-resolution images of UmuC-mKate2 and LacY-eYFP

Super-resolution reconstructions (e.g. PALM images) require that fluorescent protein molecules are observed one at a time as spatially separate foci. For typical, densely labeled samples this requires the use of a photoswitchable fluorescent protein. Because of the low cellular levels of UmuC-mKate2 in the wild-type (*recA* ^*+*^ *lexA* ^*+*^) cells, however, it was possible to reconstruct super-resolution images without photoswitching. For LacY-eYFP we used a strategy described previously [56], driving molecules into a non-bleached dark state before imaging spontaneous single-molecule returns to the fluorescent state (4000 × 34 ms frames).

To reconstruct images we identified single-molecule foci by applying a discoidal averaging filter [57] to the movies and selecting those peaks above a defined threshold value. Returning to the untreated movies, we then fit each focus with a 2D Gaussian function in order to accurately determine their center positions (non-linear least squares fitting with Levenberg-Marquadt algorithm). We reconstructed images by drawing each position as 2D Gaussian-shaped spots as described by the formula:

$$f\left(x,y\right)=\frac{1}{2\pi {\sigma_{x}\sigma }_{y}}e^{-\left(\frac{x^{2}}{2\sigma_{x}^{2}}+\frac{y^{2}}{2\sigma_{y}^{2}}\right)}$$

In this way the volume under each spot remained constant, whereas the width was determined by the fitting error in x and y (σ_x_ and σ_y_).

### Determining cellular concentrations of UmuC-mKate2

It is possible to determine the number of molecules of a fluorescent protein in a cell by dividing its total fluorescence by the average signal arising from individually measured single molecules. When measuring cells that only contain a small number of fluorescent proteins, however, individual fluorescence images tend to contain a lot of noise. We therefore decided on a different strategy for analysis of our time-sampling data. Having removed contributions from background fluorescence, we fit the loss of fluorescence signal due to photobleaching of mKate2 with an exponential decay function and used the fit amplitudes as a measure for the initial fluorescence. In this way, the total fluorescence of the cell is derived from measurements over all 296 frames of the movie, reducing the contribution of noise dramatically. The concentration of UmuC-mKate2 could then be determined for each cell using its volume as calculated from its corresponding bright field image.

For each all fluorescence movies we first flattened the images to correct for uneven illumination profile of the excitation light. To produce and of the illumination beam we averaged together each slice from all 70 movies recorded during a 3h UV-damage experiment, smoothed the resulting image by Gaussian blurring (10 pixel radius) and normalised the intensity as a fraction of the brightest pixel. This showed that illumination was already relatively flat (at the corners of the image was 70% as intense as that at the centre of the image). We then divided each movie by this “beam” movie.

For each cell we measured the change in mean fluorescence per pixel due to photobleaching. The raw data included contributions not only from UmuC-mKate2, but also cellular autofluorescence (**S1A Fig**) and background fluorescence from the glass coverslip (**S1B Fig**), which were removed prior to quantitating mKate2 fluorescence. Under our conditions, mKate2 follows a single-exponential photobleaching decay with time constant t ≈ 5 s (**S1C Fig**). We quantified the total mKate2 fluorescence of individual cells by fitting the amplitude of each decay curve. We note that by focussing on the middle portion of the cell, signal arising from the top and bottom of the cell may not be completely captured and thus measured signals may be slightly underestimated. Cell outlines were assigned using MicrobeTracker [58].

To calibrate our measurements for number of UmuC-mKate2 molecules per cell, we measured the fluorescence signals of individual molecules. To do this, we examined the final 50 frames of our movies. Here most of the cellular fluorescence had been bleached, however individual molecules could occasionally be observed as foci as they spontaneously returned to a bright state. We fit these foci with 2D Gaussian functions and made a histogram of the integrated volumes under each curve (**S1D Fig**). We could then calibrate our cellular measurements by dividing the total fluorescence of each cell by the mean value for single molecules.

#### Imaging and analysis of cells grown in shaking culture

Cells were grown in shaking culture at 37°C in EZ rich defined medium (Teknova) that included 0.2% (w/v) glucose. To induce DNA damage cells were grown until reaching mid-log phase (OD_600_ ~ 0.5), placed between two quartz sheets separated by 170 μm spacers and irradiated with 254 nm UV light from a mercury lamp (UVP) at a fluence of 10 J.m^-2^. Cells were then put back into shaking culture in preparation for imaging.

Microscopy samples were prepared by transferring 30 μl of cell suspension onto an APTES-treated coverslip and placing a clean coverslip on top. The sample was allowed to stand for 1–2 min, before excess medium was removed by gently pressing down on the top coverslip. Imaging was started within 3 min. For each sample, 10 fields were imaged taking 5–10 min in total. Cells grew and divided at similar rates before and after imaging.

In order to characterize the spatial distributions of UmuC-mKate2 foci we produced maps of their cellular locations. We achieved this by identifying every focus that presented during a movie and pinpointing its centre position using 2D Gaussian fitting. The position of each focus was then mapped to its location along the long and short axes of the cell using MicrobeTracker [58]. For each time point of our experiment, we produced a “location map” by displaying the cellular locations of all foci from all cells as Gaussian-shaped spots on a “cell” of average width and length for that time point. To correct for variation in sample size, the intensity of each resulting image was then divided by the total number of cells analyzed.

#### Analysis of fluorescent protein distribution by autocorrelation and cross-correlation

To analyze the distribution of UmuC-mKate2 in time-lapse experiments we measured autocorrelation functions across the horizontal axis of the images, which was perpendicular to the direction of flow. Images were subjected to peak filtering prior to analysis [57]. As cellular UmuC-mKate2 levels were low, foci were sparsely distributed in images. We therefore averaged along the vertical axis of each image in 20 μm wide bins. Autocorrelation was measured over a 1 μm distance lag range for each bin in each image and the resulting autocorrelation functions averaged. These autocorrelation functions were plotted as a function of time after UV exposure as a 2D contour plot. Each autocorrelation function was normalized against the mean cellular fluorescence intensity (I), such that the autocorrelation value at zero lag and I_max_ = 1. Cross-correlation functions were determined similarly, except that images of both DnaX-YPet and UmuC-mKate2 were used as input and no intensity-based normalization was used. All correlations were measured using the *xcorr* function in Matlab.

## Supporting Information

S1 Fig Western blot analysis showing proteins that cross-react with UmuC antibodies and levels of MMS-induced mutagenesis in *recA* ^+^ *lexA* ^+^ strain. (A) Proteins that interact with the affinity purified UmuC antibodies. Whole cell extracts from RW578 (Δ*umuDC*) and RW574 (*umuDC* ^+^) were separated by SDS-PAGE and subjected to western blot analysis using affinity purified polyclonal UmuC antibodies. The image clearly shows a strong signal of the expected size of UmuC in the *umuDC* ^+^ strain. However, there are also fainter bands visible both above and below the UmuC protein. All three proteins are non-specific cross-reacting proteins and not UmuC degradation products (for the faster migrating proteins) since they are also observed in WCEs from isogenic the Δ*umuDC* strain, RW578. (B) Levels of mutagenesis promoted by *umuDC* ^+^ and *umuD* ^+^ *umuC*-mKate2 in a *recA* ^+^ *lexA* ^+^ strain (RW118 and RW1480). As expected, given the multiple levels of regulation imposed on the Umu proteins to keep their intracellular levels to a minimum, the extent of *umu*-dependent mutagenesis were generally lower in a *recA* ^+^ *lexA* ^+^ background than in a *recA*(E38K) lexA(Def) strain background (**Fig 1D**) where much of the transcriptional, posttranslational and proteolytic regulation is circumvented. Similar to the *recA*(E38K) lexA(Def) strain background, the umuc-mKate2 construct gave lower levels of mutagenesis compared to wild-type UmuC, but this is entirely consistent with the lower steady state levels of the chimeric fusion protein.
(TIF)

S2 Fig Determination of cellular UmuC-mKate2 levels (EAW282). The number of molecules is determined by measuring the total fluorescence within each cell and dividing by the mean intensity of a single-molecule. Cellular intensities are determined using the fitted amplitudes of photobleaching curves rather than individual images. Contributions due to autofluorescence and background fluorescence are removed prior to fitting. (A) Change in mean fluorescence signal during photobleaching of 195 wild-type MG1655 cells used to determining autofluorescence. This mean value was subtracted from the bleaching trajectories of UmuC-mKate2 expressing cells. The autofluorescence signal was quantified by fitting with a single-exponential decay function (red line). (B) Bleaching of flow-cell background signal within a single field-of-view. Background signal from each field of view was removed from the bleaching trajectories of all cells within that field of view. The background signal was quantified by fitting with a single-exponential decay function (red line). (C) Bleaching of UmuC-mKate2 fluorescence within a single cell, corrected for background and cellular autofluorescence. The total mKate2 signal was quantified by fitting with a single-exponential decay function (red line). (D) Measurement of single molecule intensity by analysis of step-wise photobleaching trajectories. Mutasome foci were identified within time-sampling images of *recA*(E38K) cells by making average projections of movies. Intensity vs. time trajectories were measured for each focus as it photobleached, locally subtracting background fluorescence around each focus. Trajectories showed step-wise intensity transitions corresponding to photobleaching of DNA-bound UmuC-mKate2 molecules. These transitions were fit by change-point analysis [59,60]. A histogram of the step sizes of intensity transitions for 1690 trajectories, representing the intensities of single molecules, showed a relatively narrow distribution (red columns). The peak intensity (7500 arbitrary units) was taken to be the mean intensity of single molecules for calculation of UmuC-mKate2 concentrations. The initial intensities of foci prior to photobleaching produced a similarly narrow peak with a peak intensity corresponding to the single molecule intensity, indicating that pol V acts as a monomer.
(TIF)

S3 Fig Quantification of UmuC-mKate2 levels in cells (EAW282) periodically sampled from shaking culture. (A) Mean number of molecules per cell versus time. (B) Concentration of UmuC-mKate2 for shaking-culture cells, determined using the number of molecules per cell (panel A) and the cell volume measured from bright-field images. Cells were grown in EZ medium with glucose at 37°C. The entire 0.5 ml culture was irradiated with 10 J/m^2^ UV light while sandwiched between two quartz plates then returned to shaking culture. Aliquots were taken every 10 min, placed on an APTES-treated coverslip, closed in with a second plain glass coverslip and imaged. 96 × 34 ms frames were recorded using 568 nm excitation light at a power of 1800 W/cm^2^.
(TIF)

S4 Fig Control experiments showing that membrane localization of UmuC-mKate2 is a property of UmuC. (A) When expressed at extremely low levels (MG1655) from a pBAD vector (leaky expression), mKate2 presents a slight background of membrane-associated foci (top left panel), however no such foci are observable when the mKate2 levels are increased to ~ 4 nM by adding 0.01% arabinose for 2 h (top right panel). To determine if the observed membrane-localization of UmuC-mKate2 is a property of UmuC or of mKate2, we compared RW1360 expressing UmuC and cells expressing membrane-associated UmuC-mKate2 (*umuD*(K97A) *lexA*(Def) *sulA* ^-^) in the presence of ~ 6 nM mKate2 expressed from pBAD (second row of panels). (B) Plotted positions of detected foci across the short axis of the cell as histograms. Cells expressing only free mKate2 show a typical cytosolic distribution (left panel), whereas cells also expressing UmuC-mKate2 show a clear membrane-associated population (right panel).
(TIF)

S5 Fig Examples of images derived from time-sampling measurements showing tendency for UmuC-mKate2 to accumulate at the cell membrane 90 min after UV irradiation and to be localized in the cytosol 180 min after irradiation (EAW282). The images shown are composites of raw fluorescence images and peak-filtered fluorescence images. The intensity range used for each channel was the same for all composites.
(TIF)

S6 Fig Autocorrelation analysis of LacY-eYFP and DnaX-YPet. Cells (EAW191 with pBAD-LacY-eYFP or EAW282) were grown at 37°C in flow cells and irradiated *in situ* with 10, 30 or 100 J.m^-2^ of UV light (λ = 254 nm). Cells containing pBAD-LacY-eYFP were grown in EZ medium containing 0.2% (v/v) glycerol, 100 μg/ml ampicillin and 3 μg/ml L-arabinose, rather that EZ glucose. Following irradiation fluorescence images were recorded every 5 min for 180 min. Autocorrelation analysis is presented as a 2D contour plot. Blue areas indicate low correlation, whereas red areas indicate high correlation. (A) Analysis of LacY-eYFP UmuC-mKate2 cells. Clear cross-peaks at lag = ± 0.6μm appear throughout the time-lapse measurement as a result of the membrane-localized LacY-eYFP signal. (B) Analysis of DnaX-YPet UmuC-mKate2 cells. No cross-peaks are visible at lag = ± 0.6μm, consistent with the cytoslic/nucleoid associated localization of DnaX-YPet signals. A peak-enhancing filter was applied to fluorescence images prior to autocorrelation analysis [57].
(TIF)

S7 Fig Monitoring the redistribution of UmuC-mKate2 in response to UV-induced DNA damage in cells grown in shaking culture (EAW282). (A) Graphical overview of method for creating focus location maps. (B) Location maps of UmuC-mKate2 foci versus time after UV irradiation. Cells were grown in EZ medium with glucose at 37°C. The entire 0.5 ml culture was irradiated with 10 J/m^2^ UV light while sandwiched between two quartz plates then returned to shaking culture. Aliquots were taken every 10 min, placed on an APTES-treated coverslip, closed in with a second plain glass coverslip and imaged. 96 ×34 ms frames were recorded using 568 nm excitation light at a power of 1800 W/cm^2^.
(TIF)

S8 Fig Quantitation of UmuC-mKate2 in the *recA*(E38K) background (EAW307) based on time-sampling measurements. Cells were grown in EZ medium with glucose at 37°C. The entire 0.5 ml culture was irradiated with 10 J/m^2^ UV light while sandwiched between two quartz plates then returned to shaking culture. Aliquots were taken every 10 min, placed on an APTES-treated coverslip, closed in with a second plain glass coverslip and imaged. 96 × 34 ms frames were recorded using 568 nm excitation light at a power of 1800 W/cm^2^. (A) Number of molecules per cell. (B) Cellular concentrations of UmuC-mKate2.
(TIF)

S9 Fig Levels of mutasome-replisome co-localization for cells (EAW282/EAW307) grown in shaking culture. Average projections were made from the mKate2 fluorescence movies and spots persisting for ~ 300 ms were identified as mutasome foci. Replisomes were imaged using 514 nm excitation light at a power of 60 W/cm^2^, with an exposure time of 500 ms. Mutasome and replisome foci were considered co-localized if found to be within 100 nm of each other.
(TIF)

S1 Movie Movie (96 × 34 ms frames) showing UmuC-mKate2 fluorescence in wild-type (*recA* ^+^) cells (EAW282), 60 min after irradiation with UV light. (MP4)
