# Supplementary figures and images for: Regulation of Mutagenic DNA Polymerase V Activation in Space and Time
Source: PLoS Genet. 2015 Aug 28;11(8):e1005482. doi: 10.1371/journal.pgen.1005482 (PMC4552617; doi:10.1371/journal.pgen.1005482)

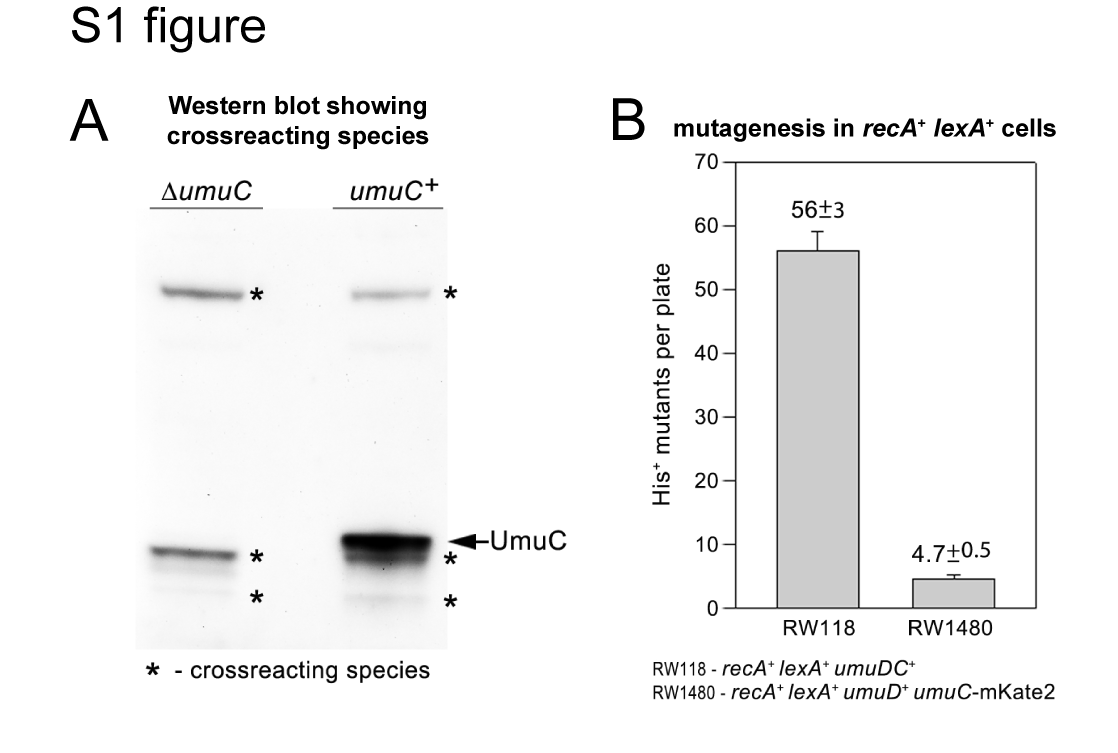

Supplement: S1 Fig — (A) Proteins that interact with the affinity purified UmuC antibodies. Whole cell extracts from RW578 (ΔumuDC) and RW574 (umuDC +) were separated by SDS-PAGE and subjected to western blot analysis using affinity purified polyclonal UmuC antibodies. The image clearly shows a strong signal of the expected size of UmuC in the umuDC + strain. However, there are also fainter bands visible both above and below the UmuC protein. All three proteins are non-specific cross-reacting proteins and not UmuC degradation products (for the faster migrating proteins) since they are also observed in WCEs from isogenic the ΔumuDC strain, RW578. (B) Levels of mutagenesis promoted by umuDC + and umuD + umuC-mKate2 in a recA + lexA + strain (RW118 and RW1480). As expected, given the multiple levels of regulation imposed on the Umu proteins to keep their intracellular levels to a minimum, the extent of umu-dependent mutagenesis were generally lower in a recA + lexA + background than in a recA(E38K) lexA(Def) strain background (Fig 1D) where much of the transcriptional, posttranslational and proteolytic regulation is circumvented. Similar to the recA(E38K) lexA(Def) strain background, the umuc-mKate2 construct gave lower levels of mutagenesis compared to wild-type UmuC, but this is entirely consistent with the lower steady state levels of the chimeric fusion protein. (TIF) [file pgen.1005482.s001.tif]

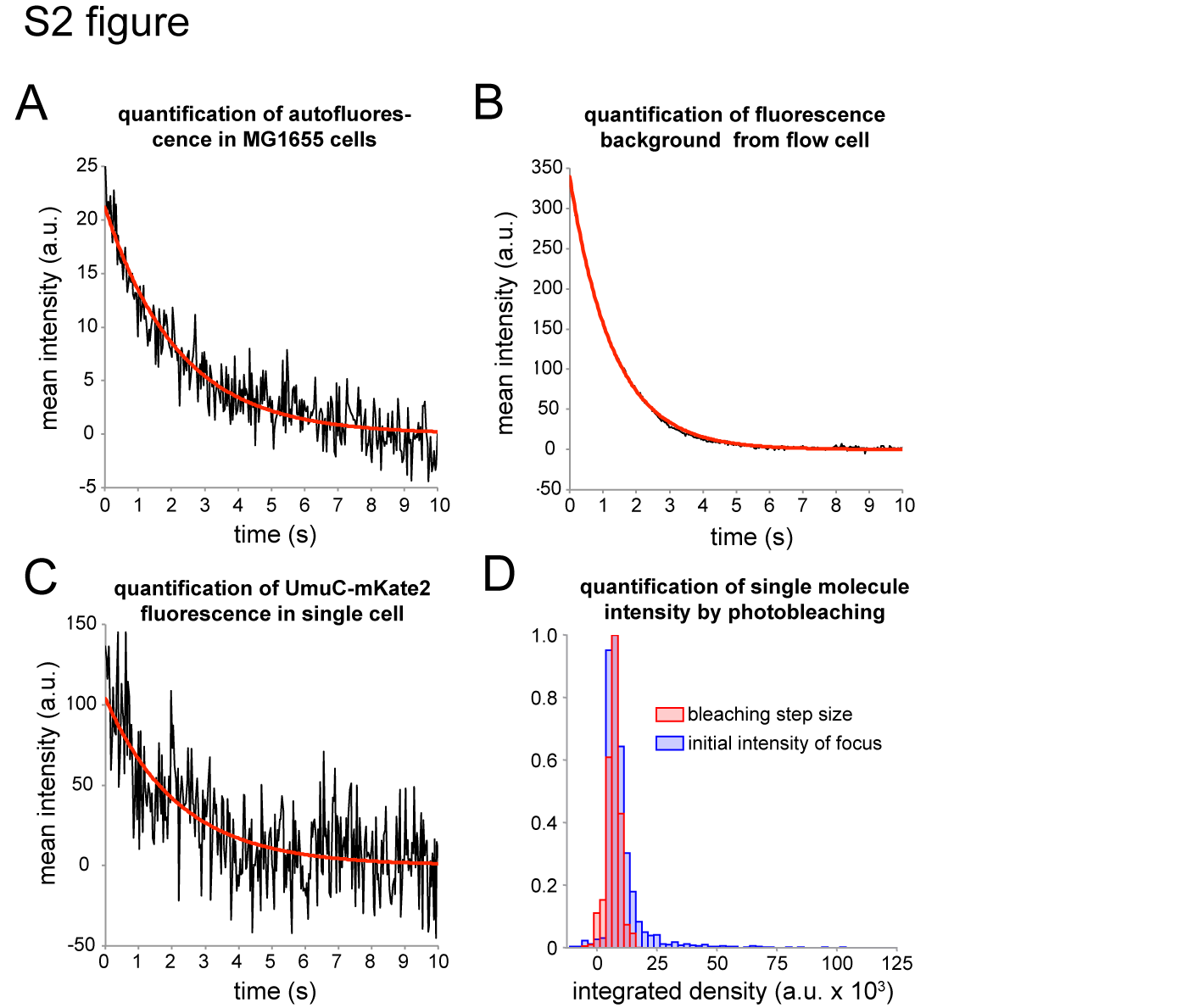

Supplement: S2 Fig — The number of molecules is determined by measuring the total fluorescence within each cell and dividing by the mean intensity of a single-molecule. Cellular intensities are determined using the fitted amplitudes of photobleaching curves rather than individual images. Contributions due to autofluorescence and background fluorescence are removed prior to fitting. (A) Change in mean fluorescence signal during photobleaching of 195 wild-type MG1655 cells used to determining autofluorescence. This mean value was subtracted from the bleaching trajectories of UmuC-mKate2 expressing cells. The autofluorescence signal was quantified by fitting with a single-exponential decay function (red line). (B) Bleaching of flow-cell background signal within a single field-of-view. Background signal from each field of view was removed from the bleaching trajectories of all cells within that field of view. The background signal was quantified by fitting with a single-exponential decay function (red line). (C) Bleaching of UmuC-mKate2 fluorescence within a single cell, corrected for background and cellular autofluorescence. The total mKate2 signal was quantified by fitting with a single-exponential decay function (red line). (D) Measurement of single molecule intensity by analysis of step-wise photobleaching trajectories. Mutasome foci were identified within time-sampling images of recA(E38K) cells by making average projections of movies. Intensity vs. time trajectories were measured for each focus as it photobleached, locally subtracting background fluorescence around each focus. Trajectories showed step-wise intensity transitions corresponding to photobleaching of DNA-bound UmuC-mKate2 molecules. These transitions were fit by change-point analysis [59,60]. A histogram of the step sizes of intensity transitions for 1690 trajectories, representing the intensities of single molecules, showed a relatively narrow distribution (red columns). The peak intensity (7500 arbitrary units) was taken [file pgen.1005482.s002.tif]

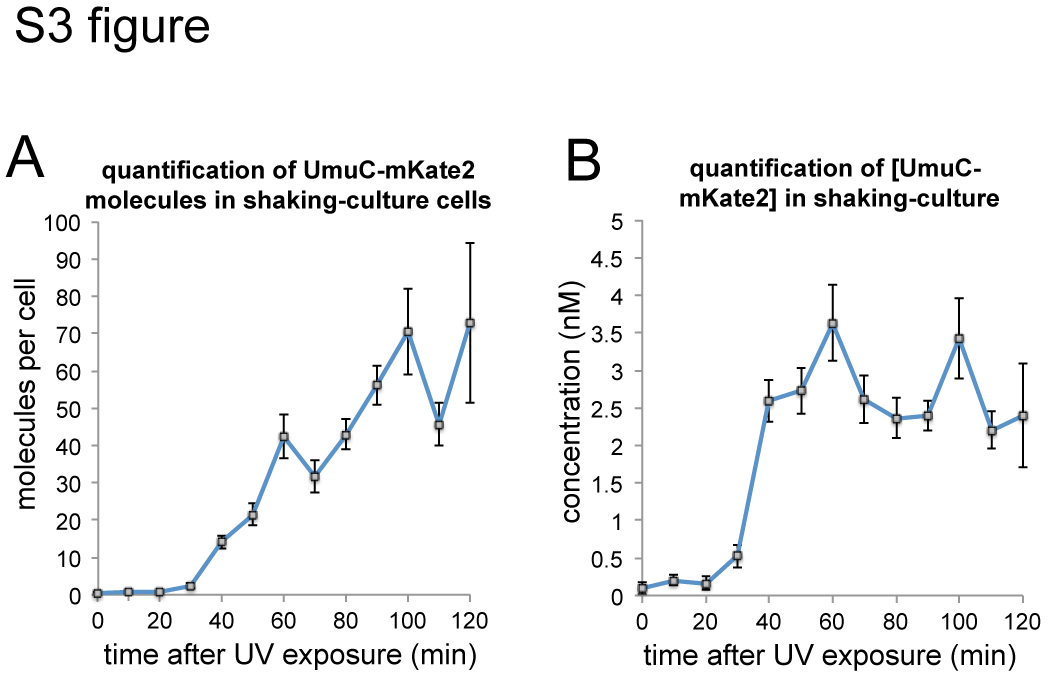

Supplement: S3 Fig — (A) Mean number of molecules per cell versus time. (B) Concentration of UmuC-mKate2 for shaking-culture cells, determined using the number of molecules per cell (panel A) and the cell volume measured from bright-field images. Cells were grown in EZ medium with glucose at 37°C. The entire 0.5 ml culture was irradiated with 10 J/m2 UV light while sandwiched between two quartz plates then returned to shaking culture. Aliquots were taken every 10 min, placed on an APTES-treated coverslip, closed in with a second plain glass coverslip and imaged. 96 × 34 ms frames were recorded using 568 nm excitation light at a power of 1800 W/cm2. (TIF) [file pgen.1005482.s003.tif]

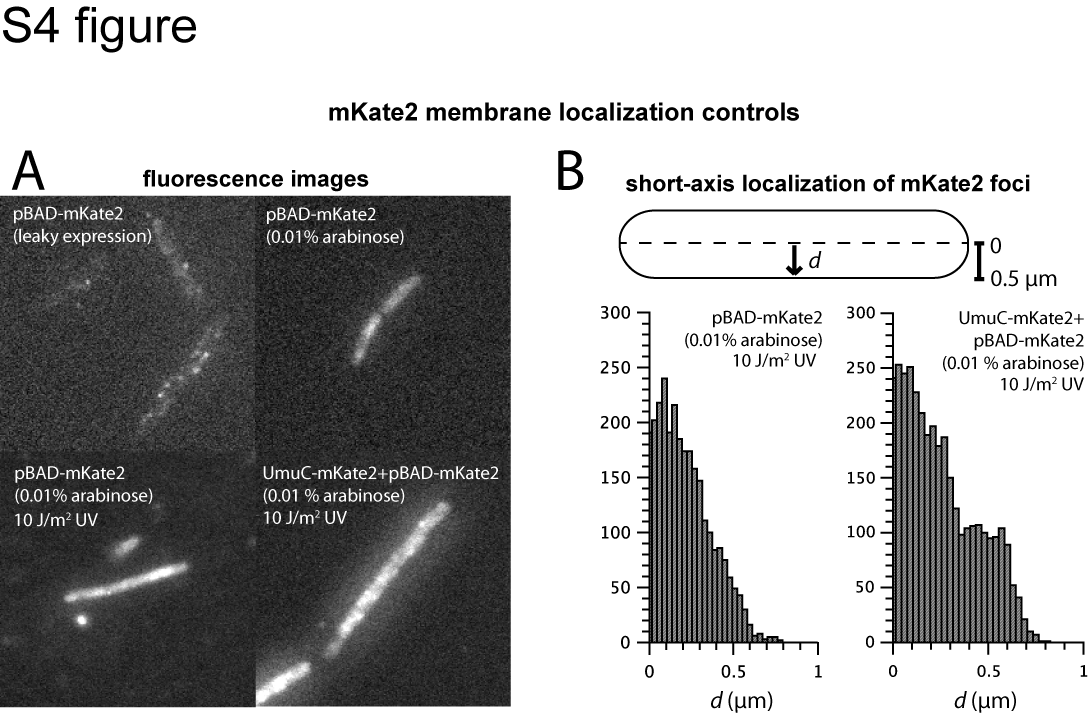

Supplement: S4 Fig — (A) When expressed at extremely low levels (MG1655) from a pBAD vector (leaky expression), mKate2 presents a slight background of membrane-associated foci (top left panel), however no such foci are observable when the mKate2 levels are increased to ~ 4 nM by adding 0.01% arabinose for 2 h (top right panel). To determine if the observed membrane-localization of UmuC-mKate2 is a property of UmuC or of mKate2, we compared RW1360 expressing UmuC and cells expressing membrane-associated UmuC-mKate2 (umuD(K97A) lexA(Def) sulA -) in the presence of ~ 6 nM mKate2 expressed from pBAD (second row of panels). (B) Plotted positions of detected foci across the short axis of the cell as histograms. Cells expressing only free mKate2 show a typical cytosolic distribution (left panel), whereas cells also expressing UmuC-mKate2 show a clear membrane-associated population (right panel). (TIF) [file pgen.1005482.s004.tif]

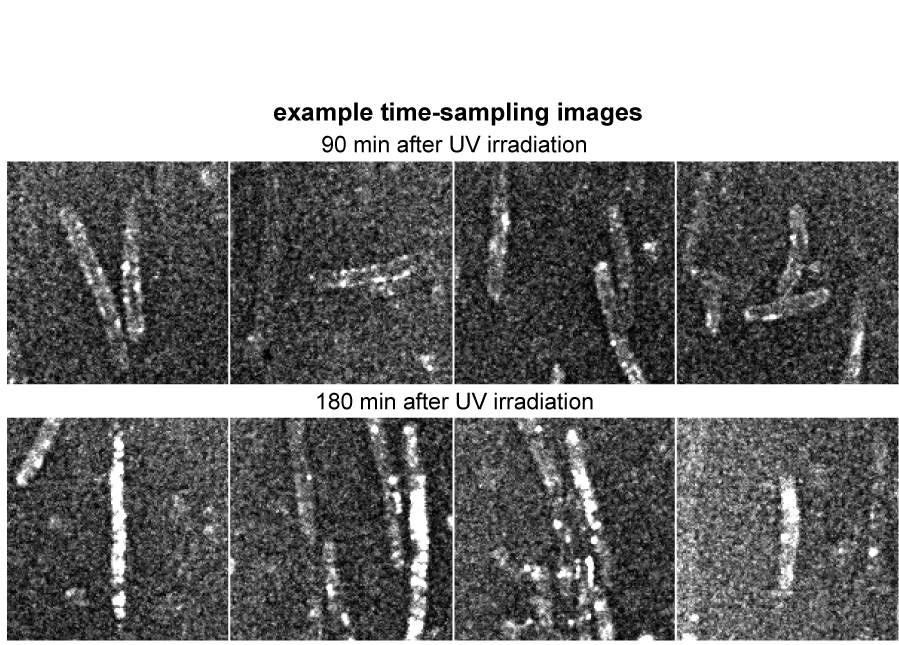

Supplement: S5 Fig — The images shown are composites of raw fluorescence images and peak-filtered fluorescence images. The intensity range used for each channel was the same for all composites. (TIF) [file pgen.1005482.s005.tif]

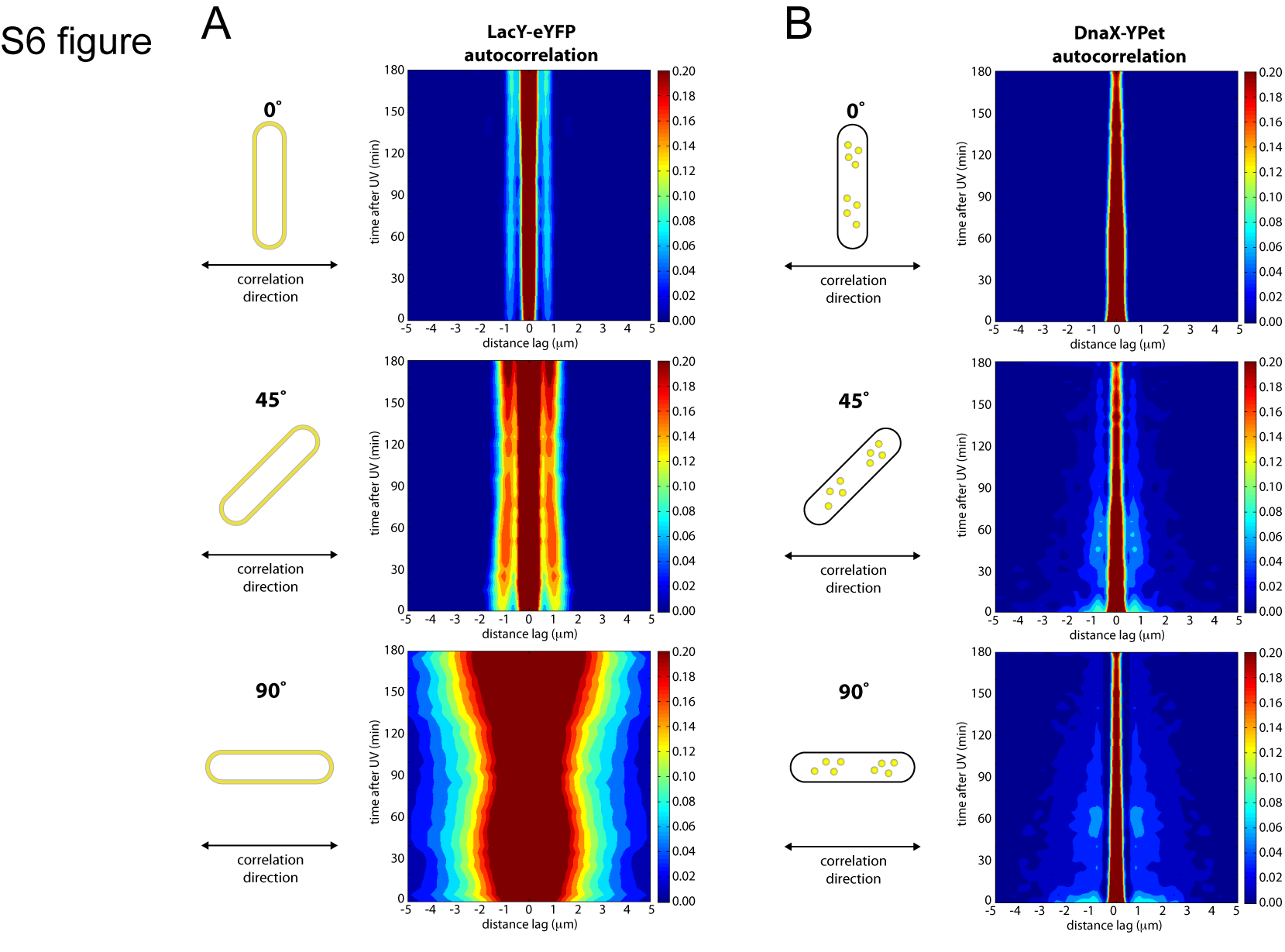

Supplement: S6 Fig — Cells (EAW191 with pBAD-LacY-eYFP or EAW282) were grown at 37°C in flow cells and irradiated in situ with 10, 30 or 100 J.m-2 of UV light (λ = 254 nm). Cells containing pBAD-LacY-eYFP were grown in EZ medium containing 0.2% (v/v) glycerol, 100 μg/ml ampicillin and 3 μg/ml L-arabinose, rather that EZ glucose. Following irradiation fluorescence images were recorded every 5 min for 180 min. Autocorrelation analysis is presented as a 2D contour plot. Blue areas indicate low correlation, whereas red areas indicate high correlation. (A) Analysis of LacY-eYFP UmuC-mKate2 cells. Clear cross-peaks at lag = ± 0.6μm appear throughout the time-lapse measurement as a result of the membrane-localized LacY-eYFP signal. (B) Analysis of DnaX-YPet UmuC-mKate2 cells. No cross-peaks are visible at lag = ± 0.6μm, consistent with the cytoslic/nucleoid associated localization of DnaX-YPet signals. A peak-enhancing filter was applied to fluorescence images prior to autocorrelation analysis [57]. (TIF) [file pgen.1005482.s006.tif]

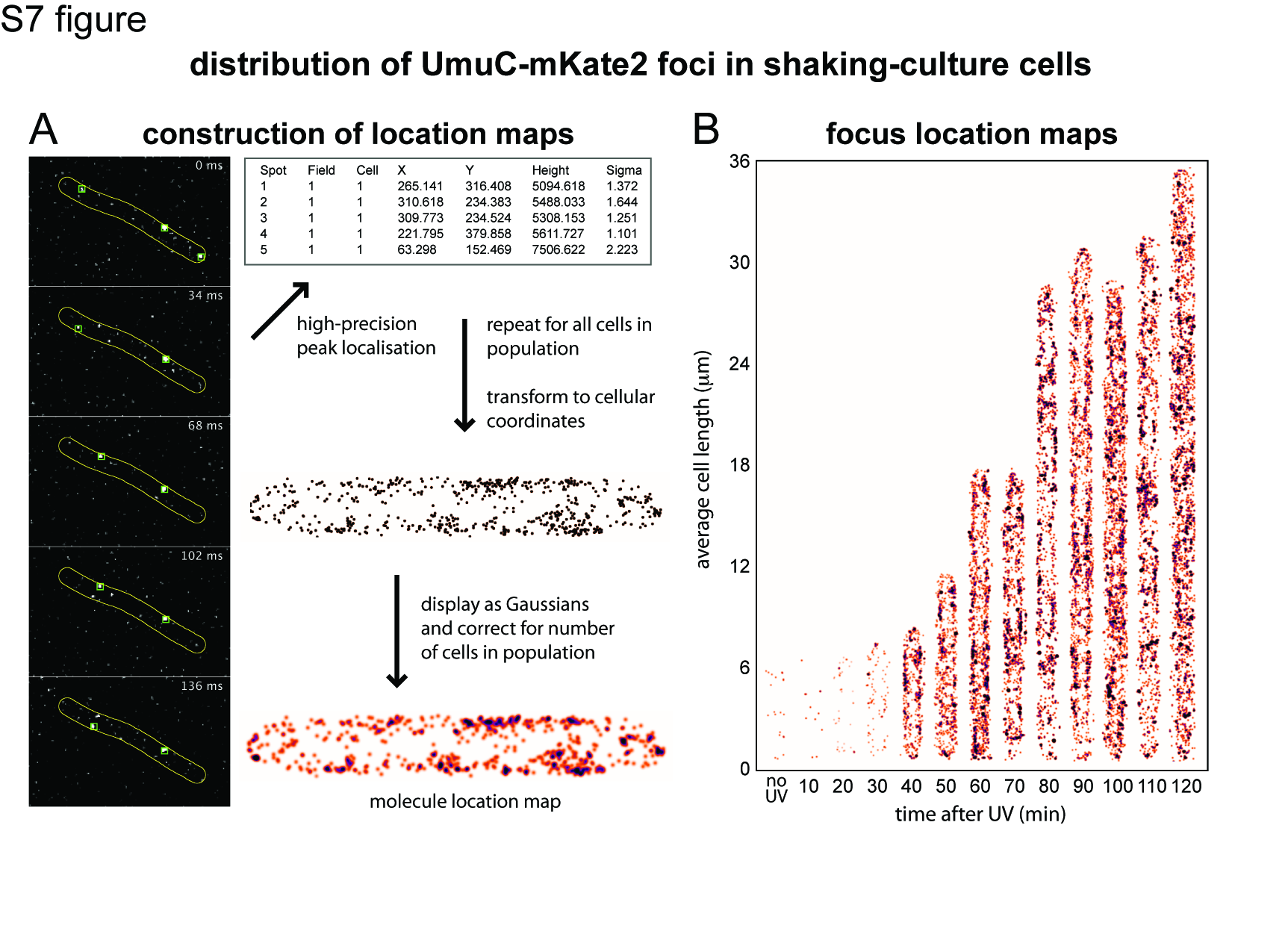

Supplement: S7 Fig — (A) Graphical overview of method for creating focus location maps. (B) Location maps of UmuC-mKate2 foci versus time after UV irradiation. Cells were grown in EZ medium with glucose at 37°C. The entire 0.5 ml culture was irradiated with 10 J/m2 UV light while sandwiched between two quartz plates then returned to shaking culture. Aliquots were taken every 10 min, placed on an APTES-treated coverslip, closed in with a second plain glass coverslip and imaged. 96 ×34 ms frames were recorded using 568 nm excitation light at a power of 1800 W/cm2. (TIF) [file pgen.1005482.s007.tif]

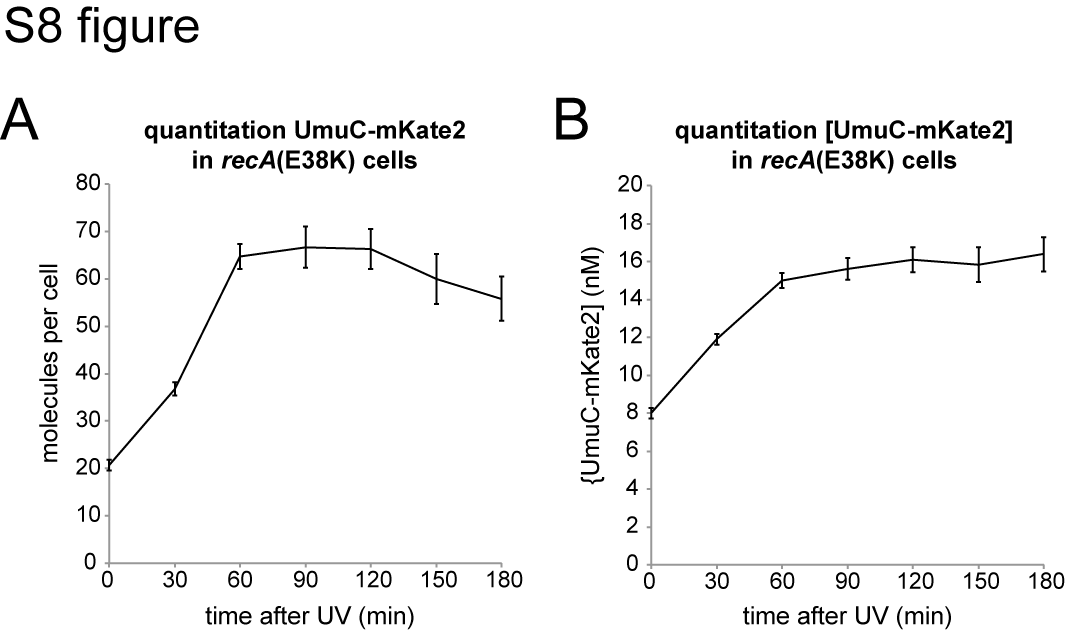

Supplement: S8 Fig — Cells were grown in EZ medium with glucose at 37°C. The entire 0.5 ml culture was irradiated with 10 J/m2 UV light while sandwiched between two quartz plates then returned to shaking culture. Aliquots were taken every 10 min, placed on an APTES-treated coverslip, closed in with a second plain glass coverslip and imaged. 96 × 34 ms frames were recorded using 568 nm excitation light at a power of 1800 W/cm2. (A) Number of molecules per cell. (B) Cellular concentrations of UmuC-mKate2. (TIF) [file pgen.1005482.s008.tif]

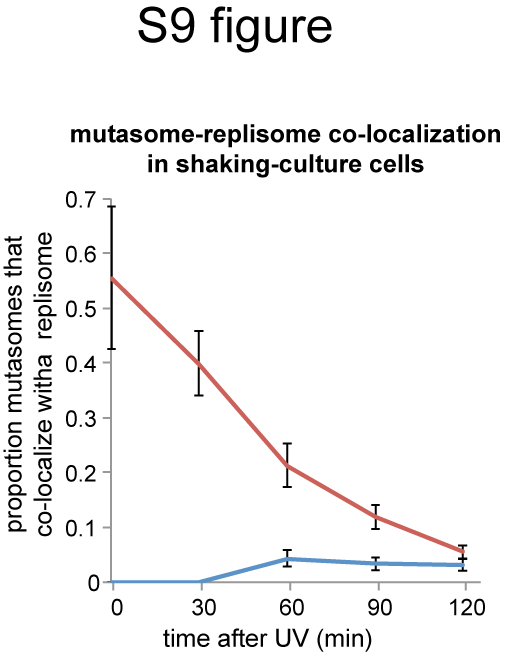

Supplement: S9 Fig — Average projections were made from the mKate2 fluorescence movies and spots persisting for ~ 300 ms were identified as mutasome foci. Replisomes were imaged using 514 nm excitation light at a power of 60 W/cm2, with an exposure time of 500 ms. Mutasome and replisome foci were considered co-localized if found to be within 100 nm of each other. (TIF) [file pgen.1005482.s009.tif]
